# Design and Synthesis of 4-(Heterocyclic Substituted Amino)-1*H*-Pyrazole-3-Carboxamide Derivatives and Their Potent Activity against Acute Myeloid Leukemia (AML)

**DOI:** 10.3390/ijms20225739

**Published:** 2019-11-15

**Authors:** Yanle Zhi, Zhijie Wang, Chao Yao, Baoquan Li, Hao Heng, Jiongheng Cai, Li Xiang, Yue Wang, Tao Lu, Shuai Lu

**Affiliations:** 1 School of Pharmacy, Henan University of Chinese Medicine, Zhengzhou 450046, China; 15311050167@stu.cpu.edu.cn; 2 School of Pharmacy, China Pharmaceutical University, 24 Tongjiaxiang, Nanjing 210009, China; 16401607@stu.cpu.edu.cn; 3 Collaborative Innovation Center for Respiratory Disease Diagnosis, Treatment & Chinese Medicine Development of Henan Province, Henan University of Chinese Medicine, Zhengzhou 450046, China; 4 School of Science, China Pharmaceutical University, 639 Longmian Avenue, Nanjing 211198, China; 3119050171@stu.cpu.edu.cn (Z.W.); 15211050685@stu.cpu.edu.cn (C.Y.); 15221051005@stu.cpu.edu.cn (B.L.); dg1924026@smail.nju.edu.cn (H.H.); 3319051009@stu.cpu.edu.cn (J.C.); 1020001155@cpu.edu.cn (Y.W.)

**Keywords:** 1*H*-pyrazole-3-carboxamide, AML, Cyclin-dependent kinases, fms-like receptor tyrosine kinase 3, protein kinase inhibitors, anti-cancer

## Abstract

Fms-like receptor tyrosine kinase 3 (FLT3) has been emerging as an attractive target for the treatment of acute myeloid leukemia (AML). By modifying the structure of FN-1501, a potent FLT3 inhibitor, 24 novel 1*H*-pyrazole-3-carboxamide derivatives were designed and synthesized. Compound **8t** showed strong activity against FLT3 (IC_50_: 0.089 nM) and CDK2/4 (IC_50_: 0.719/0.770 nM), which is more efficient than FN-1501(FLT3, IC_50_: 2.33 nM; CDK2/4, IC_50_: 1.02/0.39 nM). Compound **8t** also showed excellent inhibitory activity against a variety of FLT3 mutants (IC_50_ < 5 nM), and potent anti-proliferative effect within the nanomolar range on acute myeloid leukemia (MV4-11, IC_50_: 1.22 nM). In addition, compound **8t** significantly inhibited the proliferation of most human cell lines of NCI60 (GI_50_ < 1 μM for most cell lines). Taken together, these results demonstrated the potential of **8t** as a novel compound for further development into a kinase inhibitor applied in cancer therapeutics.

## 1. Introduction

Acute myeloid leukemia (AML) is a malignant hematopoietic disease, characterized by uncontrolled proliferation of hematopoietic progenitor cells of the myeloid lineage within the bone marrow [1]. Fms-like receptor tyrosine kinase 3 (FLT3) represents a promising target for treatment of AML [2,3]. High expression of FLT3 is a common feature of AML and acute lymphoblastic leukemia (ALL) [4]. Furthermore, FLT3 mutations occur in approximately 30% of new diagnosed AML patients and tend to have a negative prognostic effect [5,6]. Two major classes of activating FLT3 mutations have been identified in AML patients, internal-tandem duplications (ITDs) and tyrosine kinase domain (TKD) point mutations [7]. Mutation of FLT3 causes ligand-independent autophosphorylation and constitutive activation of downstream pathways, including RAS/MEK, PI3K/AKT/mTOR, and JAK/STAT. Excessive activation of these pathways always results in uncontrolled cell proliferation [8,9].

**Figure 1:**
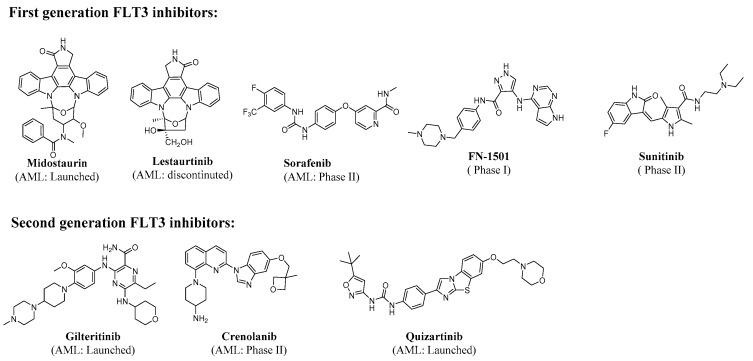
Chemical structures of several Fms-like receptor tyrosine kinase 3 (FLT3) inhibitors in clinical trials for treatment of acute myeloid leukemia (AML).

As shown in Figure 1, various small molecular FLT3 inhibitors have been developed [7,10,11,12]. As a first-generation FLT3 inhibitor, midostaurin was first approved by FDA (in April 2017) for the treatment of newly diagnosed FLT3-mutant AML in combination with chemotherapy [13]. However, other first-generation FLT3 inhibitors were characterized by low clinical efficacy and significant toxicity. Despite initial optimism, response rates and response duration of those inhibitors were limited in patients with relapsed or refractory AML, due to the FLT3 mutation and lack of sustained FLT3 inhibition in vivo [14,15,16]. Subsequently, a series of second-generation FLT3 inhibitors were developed and achieved significant therapeutic effects. Up to now, the second-generation FLT3 inhibitors gilteritinib (approved by FDA) and quizartinib (approved in Japan) have been used for the treatment of relapsed or refractory AML [17]. Crenolanib also entered Phase III clinical research in 2017 (For treatment of Newly Diagnosed FLT3 Mutated AML). As shown in Table 1, although high selectivity against FLT3 was achieved for these second-generation FLT3 inhibitors, they still inhibit other targets that are also associated with the tumorigenesis [14,18,19]. Therefore, new agents with potent and sustained inhibition of FLT3 and the related signal pathways were noted to be beneficial to the suppression of tumor cell proliferation and overcoming drug resistance.

Cyclin-dependent kinases (CDKs) are a family of serine/threonine protein kinases that are known to play a vital role in cell cycle regulation and modulating the transcription activity [20]. Cell cycle dysregulation, resulting from aberrant mitogenic signaling and leading to uncontrolled proliferation, is one of the hallmarks of cancer [21]. Thus, inhibitors that simultaneously block FLT3 and CDKs could synergistically improve the response rate and duration in the treatment of AML. For instance, FN-1501 (Figure 1) is a FLT3 and CDKs inhibitor that we have reported, showing significant anti-AML activity [22]. In this paper, we further modified the structure of FN-1501 by optimizing the moieties that bind to the hydrophobic zone and hydrophilic regions in FLT3, and then a series of compounds with better FLT3/CDKs inhibitory activities were discovered.

## 2. Results and Discussion

### 2.1. Chemistry

**Scheme 1:**
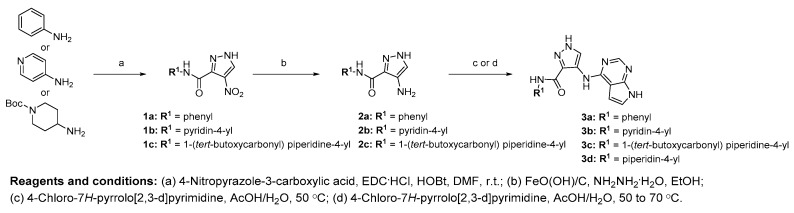
Synthesis of compounds **3a**–**3d.**

Coupling 4-nitropyrazole-3-carboxylic acid with a series of amine, followed by the reduction reaction, generated the intermediates **2a**–**2c**. Compounds **3a**–**3c** were then obtained by the substitution of **2a**–**2c** with 4-chloro-7*H*-pyrrolo[2,3-d]pyrimidine (Scheme 1). Boc group was removed from **3c** to produce **3d** in the last step.

**Scheme 2:**
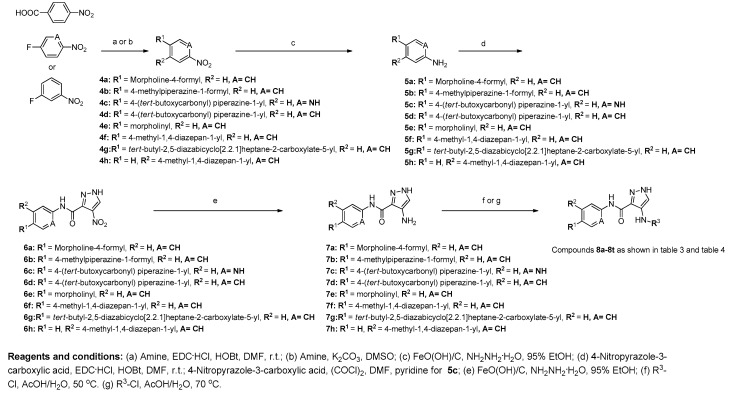
Synthesis of compounds **8a**–**8t.**

Compounds **8a**–**8t** were prepared as shown in Scheme 2. Intermediates **4a** and **4b** were prepared by coupling p-nitrobenzoic acid with the corresponding amines. Nucleophilic substitution of the corresponding amines with 5-fluoro-2-nitropyridine or 1-fluoro-4-nitrobenzene afforded the intermediates **4c**–**4h**. Then hydrogenation of the nitro group of **4a**–**4h** yielded intermediates **5a**–**5h**. Intermediates **5a**–**5h** were reacted with 4-nitro-1*H*-pyrazole-3-carbonyl to give **6a**–**6h**, followed by the reduction reaction, to yield the intermediate products **7a**–**7h**. Intermediates **7a**–**7b** and **7e**–**7h** were reacted with the appropriate chlorides to yield the desired products **8a**–**8g** and **8r**. Intermediates **7c** and **7d** were reacted with the appropriate chlorides under the appropriate temperature (initially 50 °C), and the Boc group were then removed by increasing the temperature to 70 °C when **7c** and **7d** vanished (TLC detection), to yield the desired products **8h**–**8q**, **8s**, and **8t** (Scheme 2). 

### 2.2. Structure-Activity Relationship Study

The enzymatic inhibitory activities of the target compounds were evaluated by CDK2, CDK4, and FLT3 kinase activity assays, and the cell-growth inhibitory potency against AML cell line MV4-11 were further evaluated for selected compounds (**8a**–**8t**). The results were summarized in Table 2, Table 3 and Table 4.

As shown in Table 2, compounds **3a** and **3b** were synthesized to evaluate if the hydrophilic groups were necessary for the inhibitory activity against CDK2/4 and FLT3. Their activities against CDK4 largely decreased compared with **FN-1501**, which confirmed that the hydrophilic group was important for binding to CDK4. Furthermore, replacement of benzene and pyridine rings (**3a and 3b**) with piperidine (**3c**, **3d**) caused reduction of their activity against CDK2/4 and FLT3. This suggested that both hydrophilic group and aromatic-ring structure were necessary for the compounds to inhibit CDK2/4 and FLT3.

**Figure 2:**
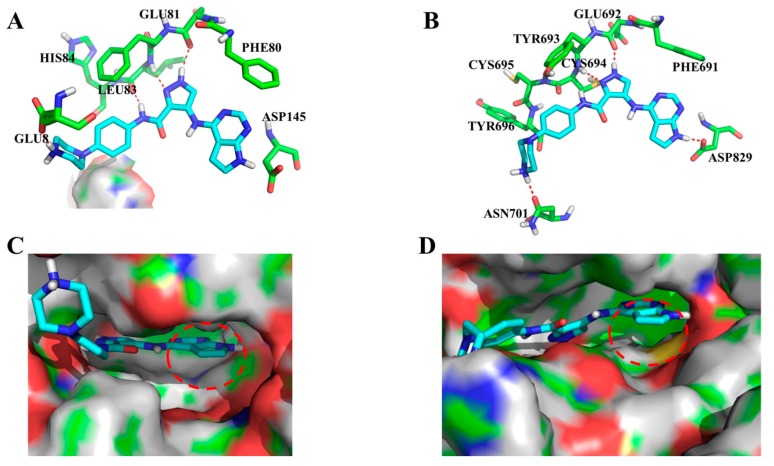
Binding mode analysis of compound **8h** bound to cyclin-dependent kinase 2 (CDK2) (PDB code 2VU3) and FLT3 (homology model). Stick model of **8h** in CDK2 (**A**) and FLT3 (**B**); Surface model of **8h** in CDK2 (**C**) and FLT3 (**D**), Red circle: the deep hydrophobic pocket

In order to find out the optimal groups in the hydrophilic region of ATP-binding site, a series of substitutions were introduced to the benzene ring (**8a**–**8j**, as shown in Table 3). A decrease of kinase inhibitory activity was observed when fixing the *N*-methylpiperazine or morpholine to benzene by carbonyl group (**8a** and **8b**) compared with FN-1501. Bulkier groups (such as homopiperazine), when directly connected to the benzene ring, generally had no obvious influence on the activities of compunds **8c** and **8d** against CDK2/CDK4 and FLT3. Changing the *N*-methylhomopiperazine (**8c** and **8d)** to *meta*-position (**8e** and **8f**) in the benzene ring caused an obvious decrease in the inhibitory activities against CDK4 and the antiproliferative activity against MV4-11. Similarly, replacement of homopiperazine (**8d**) with morpholine (**8g**) caused a 10-fold decrease of IC_50_ value against FLT3 compared with FN-1501. However, replacement of NH by S in the hydrophobic ring structure has little effect on their kinase inhibitory activity. Moreover, compound **8h** with a single piperazine group showed improved inhibitory effects against CDK2 and FLT3, probably benefiting from the extra intermolecular interaction between the secondary amine of piperazine with CDK2 (GLU8)/FLT3 (ASN701) (Figure 2A,B). Compound **8h** also exhibited high inhibitory activity against MV4-11 (IC_50_: 7.3 nM). However, the derivatives **8i** and **8j** consisted of the bridged-piperazine rings displayed weaker inhibitory effects against CDK2/4 and FLT3, when compared with compound **8h**. Overall, a piperazine ring that directly attaches to the para position of the benzene ring is beneficial in elevating the inhibitory activity against CDK2/4 and FLT3.

We next turned our attention to the aromatic linker that connected the piperazine ring and the structure bound in the hinge area. As shown in Table 4, replacement of the benzene ring with 2-pyridine (**8k**, **8l**) significantly decreased their inhibitory activities against CDK2, and attenuated the inhibitory activities against CDK4 and FLT3 moderately. Molecular modeling showed that an unoccupied space existed in the deep hydrophobic pocket (Figure 2C,D), suggesting that the additional hydrophobic substitution was beneficial to the inhibitory activity against CDK2/4 and FLT3. Therefore, we retained the pyridine ring as aromatic linker and introduced different groups in the deep hydrophobic pocket. When the five-member ring in the bicycle system was replaced by benzene ring (**8m**), the kinase inhibitory activities were basically maintained compared with **8l**. Further alteration with the saturated five-member ring (**8q**), led to overall reduction in kinase and cell inhibitory activity. However, compounds **8n**, **8o**, and **8p**, which contained the additional bulkier hydrophobic groups in different size in the deep hydrophobic pocket, showed improved inhibitory activities against CDK4 and FLT3 compared with **8k**. Unfortunately, these compounds exhibited weaker anti-proliferative effects on MV4-11 cells (IC_50_ value: 0.045–0.35 μM). It was presumed that the introduction of pyridine ring may reduce the permeability of the entire molecule, and thus decrease their activity to MV4-11. Therefore, benzene ring was reserved as the aromatic linker.Combining the bulkier hydrophobic substituents in deep pocket and the benzene as aromatic linker, compounds **8r**–**8t** were designed (as shown in Table 4). Generally, compounds **8r** and **8s** showed enhanced CDK2/4 and FLT3 inhibitory activities as well as the antiproliferative potency against MV4-11 cells, compared with compound **8n**. As the optimal compound, **8t** exhibited the sub-nanomolar IC_50_ values against CDK2 (0.719 nM), CDK4 (0.770 nM), and FLT3 (0.0890 nM), and consistently strong anti-proliferative activity in MV4-11 cells (IC_50_: 1.22 nM). Thus, the step-by-step structural optimization demonstrated that the combination of piperazine in the hydrophilic pocket, benzene ring as the aromatic linker, and the bulkier fused ring in the deep hydrophobic pocket were beneficial for kinase inhibitory activity and anti-proliferative activity to MV4-11.

### 2.3. Molecular Modeling of Compound **8t** with CDK2 and FLT3

**Figure 3:**
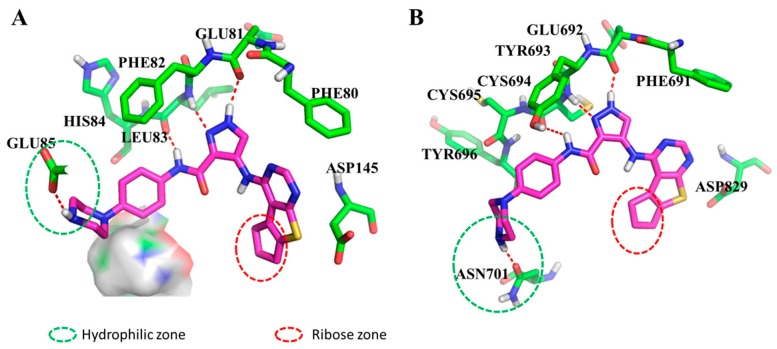
Binding mode analysis of compounds **8t** in CDK2 (**A**, PDB code 2VU3) and FLT3 (**B**, homology model).

Compound **8t** showed optimal FLT3/CDK2/CDK4 inhibitory activities. Hence, the binding mode of compound **8t** with CDK2 and FLT3 were elucidated using a docking model. Since **8t** is a type I FLT3 inhibitor, we used our homology model structure of “DFG-in” FLT3 [22]. As shown in Figure 3, compound **8t** binded to the ATP-binding site of CDK2 and FLT3 in an orientation similar to FN-1501 [22]. The pyrazole-3-carboxamide skeleton of compound **8t** formed three conserved hydrogen bonds with the hinge region of CDK2 and FLT3 respectively (Figure 3). The aromatic heterocycle moiety occupied the hydrophobic pocket and the piperazine group extended to solvent accessible area. The difference was that the NH of piperazine formed a hydrogen bond with GLU85 in CDK2 and it also formed a hydrogen bond with ASN701 in FLT3. The cyclopentane extended to ribose zones which were not occupied by FN-1501. This binding mode was beneficial to improving the inhibitory activity of **8t** against CDK2 and FLT3. In general, the docking results further confirmed the rationality of our design strategy.

### 2.4. Kinase Profiling

In order to investigate the kinase profile vulnerable to compound **8t**, its enzymatic inhibitory effects against 32 kinases were tested (Table S1), which were the representative kinase drug targets. Among those kinases (Table 5), compound **8t** showed significant inhibitory activities against CDKs and FLT3, except for CDK1 that is considered not reasonable as an anti-tumor target [20]. In addition, compound **8**t exhibited inhibitory activities against KDR/VEGFR2, ERK7, FLT1, FLT4, and GSK3β (Table 5), which were related to the tumorigenesis. These data indicated that compound **8t** is a highly potent pan-kinase inhibitor with the prominent inhibitory potency against CDKs and FLT3. Furthermore, compound **8t** potently inhibited eight FLT3 mutants (IC_50_ values less than 5 nM) that are correlated to the drug resistance [23,24]. Compound **8t** also showed significant inhibitory activity against the FLT3 (ITD)-F691L mutation (IC_50_: 0.6 nM), which led to the drug resistance to FLT3 inhibitors, such as quizartinib. Accordingly, in the BaF3 cells that are transformed with FLT3-ITD-F691L, compound **8t** showed the improved antiproliferative activity over quizartinib (Table S2).

### 2.5. In vitro Cell Assays

With these findings, we submitted compound **8t** to National Cancer Institute (NCI) to evaluate their antitumor efficacy against 60 human cancer cell lines. As shown in Table 6, compound **8t** exhibited anti-proliferative activities against a variety of cancer cell lines, which was consistent with its multi-kinase inhibition potency, indicating that **8t** has the potential of further development as a powerful anti-tumor agent for various human cancers, including AML.

### 2.6. Cellular Mode of Action

**Figure 4:**
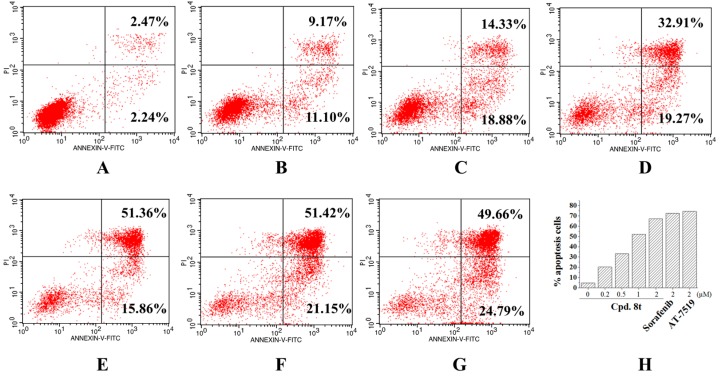
MV4-11 cells were treated with compound **8t** for 24 h and then analyzed by AnnexinV/PI staining. The percentage of cells undergoing apoptosis was defined as the sum of early apoptosis, advanced apoptosis and necrotic cells. (**A**) DMSO control; (**B**) compound **8t** at 0.2 μM; (**C**) compound **8t** at 0.5 μM; (**D**) compound **8t** at 1 μM; (**E**) compound **8t** at 2 μM; (**F**) Sorafenib at 2 μM; (**G**) AT-7519 at 2 μM; (**H**) Quantification of apoptotic cells.

To characterize the mode of cellular effects induced by compound **8t**, flow cytometry was performed in MV4-11 cell line. Since compound **8t** had strong inhibitory activity against FLT3 and CDK, sorafenib with potent inhibitory activity against FLT3 and pan-CDK inhibitor AT-7519 were selected as the positive controls [25,26]. As detected by annexin V staining, not only was the apoptosis triggered, but also a dose-dependent increase in the percentage of apoptotic and dead cells was seen (Figure 4). In the presence of vehicle alone for 24 h, only 4.7% of the cells underwent apoptosis, while treatment with compound **8t** at 2 μM for 24 h led to an apoptosis rate up to 51.36%.

**Figure 5:**
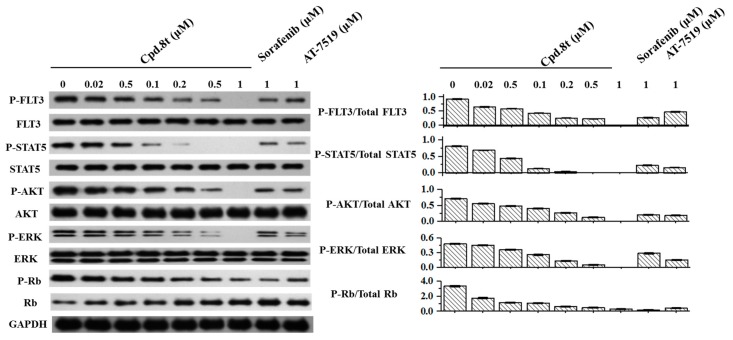
MV4-11 cells were treated with compound **8t**, AT-7519, or sorafenib for 4 h, and the phosphorylation of FLT3, STAT5, ERK, AKT, and Rb protein was analyzed by immunoblotting.

To further investigate whether the antitumor activities were relevant to the inhibition of FLT3 and CDK2, we then examined the relative signaling proteins in MV4-11 cells treated with compound **8t** by western blot. According to the MV4-11 cell-based western blot assays, the phosphorylation of the FLT3 was weakened by compound **8t** in a dose-dependent manner, and eliminated when the compound concentration was increased to 1 μM (Figure 5), which exhibited more potent inhibition effects than sorafenib. As downstream signal pathways of FLT3, the phosphorylation of STAT5/AKT/ERK were also completely blocked at 1 μM. Compound **8t** also inhibited the phosphorylation of the retinoblastoma protein (Rb), a key downstream factor of CDK2/4, in a dose-dependent manner. At the concentration of 1 μM, compound **8t** exhibited comparable inhibitory effects against the phosphorylation of Rb with AT-7519. In all, the anti-proliferative activity of compound **8t** was associated with the inhibition of FLT3 and CDK2/4.

## 3. Materials and Methods

Unless otherwise specified, reagents were purchased from commercial suppliers and used without further purification. Melting points were determined by X-4 digital display micro-melting point apparatus (Tech Instrument Co., Ltd., Beijing, China); NMR spectra were recorded on Bruker AVANCE AV-600 spectrometer (600 MHz for ^1^H, 150 MHz for ^13^C) or Bruker AVANCE AV-300 spectrometer (300 MHz for ^1^H, 75 MHz for ^13^C); Mass spectra were obtained on the Agilent 1100 LC/MSD mass spectrometer (Agilent, Santa Clara, CA, USA). All reactions were monitored by TLC (Merck Kieselgel GF254, Merck, Kenilworth, NJ, China) and spots were visualized with UV light or iodine. The purity of biologically evaluated compounds was >95% as determined by HPLC.

### 3.1. Procedure A For the Synthesis of Compounds **4a** and **4b**

The mixture of appropriate amine (18.5 mmol), p-nitrobenzoic acid (20.4 mmol), EDC (22.2 mmol), HOBT (22.2 mmol) in DMF (30 mL) was stirred for 24 h. The ice water (100 mL) was added to the reaction mixture. A large amount of yellow solid precipitation (compounds **4a** and **4b**) was acquired. Compounds **4a** and **4b** were used without further purification.

### 3.2. Procedure B For the Synthesis of Compounds **4c**–**4h**

Fluorobenzene or fluoropyridine (46.3 mmol) and K_2_CO_3_ (69.5 mmol) were dissolved in DMSO (50 mL). The reaction mixture was stirred at r.t. for 30 min and then amine (69.5 mmol) was added. The reaction mixture was stirred at 70 °C for 5 h. The ice water (500 mL) was added to the reaction mixture. A large amount of yellow solid precipitation (compounds **4c**–**4h**) was acquired. Compounds **4c**–**4h** were used for further reaction without purification.

### 3.3. Procedure C For the Synthesis of Compounds **2a**–**2c**, **5a**–**5h**, and **7a**–**7h**

To a suspension of compounds **1a**–**1c**, **4a**–**4h**, **6c**–**6h**, or **4a**–**4h** (26.2 mmol) in 95% ethanol (100 mL), 85% NH_2_NH_2_^.^H_2_O (262 mmol), 95% ethanol (100 mL), and iron (III) oxide hydroxide (FeO(OH)/C, 0.5 g) were added and heated to reflux. When TLC analysis showed complete conversion of the starting material, the reaction mixture was filtrate through Celite^®^ and the filtrate was concentrated in vacuum. The crude product was purified by silica gel column chromatography (DCM/MeOH) to yield the title compound as white solid.

### 3.4. Procedure D for the Synthesis of Compounds **1a**–**1c** and **6a**–**6h**

4-nitro-1*H*-pyrazole-3-carboxylic acid (4.19 g, 13.94 mmol) was dissolved in 20 mL THF, DMF (0.5 mL) and oxalyl chloride (1.78 mL, 20.91 mmol) were added at 0 °C, the resultant mixture was stirred at room temperature for 60 min. After the mixture was concentrated in vacuo, the residue was dissolved in pyridine (20 mL), and the solution was added dropwise into the solution of 7 (dissolved in 20 mL pyridine) at 0 °C. The solution was stirred for 6 h at 25 °C. Upon completion of the reaction, the solvent was removed on a rotary evaporator. Then water (100 mL) was added, and the mixture was basified using 10% NaOH until pH 8~9. The solid precipitation was filtered to give the crude product, which was used for next step without further purification.

### 3.5. Procedure E for the Synthesis of Compounds **3a**–**3c**, **8a**–**8g**, and **8r**

Compounds **2a**–**2c**, **7a**–**7b**, or **7e**–**7h** (10 mmol) were reacted with corresponding chlorides (12 mmol) in AcOH/H_2_O:1/1 (10 mL) at 50 °C. When TLC analysis showed complete conversion of the starting material, 10% NaOH was added and the pH was adjusted to 8–9. The precipitate was collected and purified by silica gel column chromatography with DCM/MeOH (30/1) to yield the compounds **3a**–**3c**, **8a**–**8g** and **8r**.

### 3.6. Procedure F for the Synthesis of Compounds **3d**, **8h**–**8q** and **8s**–**8t**

Compounds **3c**, **7c**–**7d**, or **7g** (10 mmol) were reacted with corresponding chlorides (12 mmol) in AcOH/H_2_O:1/1 (10 mL) at 50 °C. TLC analysis showed complete conversion of **2c**, **7c**–**7d**, or **7g**, increasing the reaction temperature to 70 °C (5 h) to the cleavage of *t*-butylcarbamoyl group. Upon completion of the reaction, 10% NaOH was added and the pH was adjusted to 8–9. The precipitate was collected and purified by silica gel column chromatography with DCM/MeOH (30/1) to yield the compounds **3d**, **8h**–**8q**, and **8s**–**8t**. 

Detailed synthetic process and structural characterization were provided in the Supplementary Materials.

### 3.7. Kinase Inhibition Assay

Activities of kinases were determined using Hot-SpotSM kinase assay which was performed by Reaction Biology Corp. (Malvern PA, USA) as described previously [27].

### 3.8. Cell Growth Inhibition Assay

The human AML cell line MV4-11 was purchased from the American Type Culture Collection (ATCC) (Manassas, VA, USA). MV4-11 was cultured in IMDM media (Corning, Crown Bioscience Inc., Taicang, China) with 10% FBS and supplemented with 2% L-glutamine and 1% pen/strep. The MV4-11 cell line was maintained in culture media at 37 °C with 5% CO_2_. The effects of target compounds on MV4-11 proliferation was performed by Crown Bioscience Inc. Cells were grown in 96-well culture plates (10,000/well). The compounds of various concentrations were added into the plates. Cell proliferation was determined after treatment with compounds for 72 h. Cell viability was measured using the CellTiter-Glo assay (Promega, Crown Bioscience Inc., Taicang, China) according to the manufacturer’s instructions, and luminescence was measured in a multilabel reader (Envision2014, PerkinElmer, Crown Bioscience Inc., Taicang, China). Data were normalized to the control group (DMSO) and represented by the mean of three independent measurements with standard error of <20%. IC_50_ values were calculated using Prism 5.0 (GraphPad Software, San Diego, CA, USA).

Analysis of pFLT3, pSTAT5, pERK, pAKT, and pRb in vitro: to determine the levels of pFLT3, pSTAT5, pERK, pAKT, and pRb, cells were seeded in a 6-well cell culture plate at a density of 400,000 cells per well for MV4-11 in a total volume of 1800 μL and incubated overnight in medium containing 10% fetal bovine serum (Life Technologies, Rockville, MD, USA). Then 200 μL of serially diluted compounds were added to each well the next day. Cell lysates were harvested after 4 h and pFLT3, pSTAT5, pERK, pAKT, or pRb were quantified using assay kits for FLT3/Phospho-FLT3Tyr589/591, pSTAT5 (Tyr694)/total STAT5, pRb (Ser807)/total Rb (Nanjin keyGen Biotech Company, Nanjing, China) following the manufacturer’s protocols.

### 3.9. Cell Apoptosis Assay

The apoptosis of MV4-11 cells was determined by Annexin V-FITC/PI assay. Annexin V binds to phosphatidylserine, which is exposed on the cell membrane and is one of the earliest indicators of cellular apoptosis. PI (Propidium Iodide) is used as a DNA stain for both flow cytometry to evaluate cell viability or DNA content in cell cycle analysis and microscopy to visualize the nucleus and other DNA containing organelles. It can be used to differentiate necrotic, apoptotic, and normal cells. Cells (2 × 10^5^) were seeded in 6-well plate and were treated with varying concentrations of inhibitor for 24 h. MV4-11 cells were collected and incubated with FITC-conjugated Annexin V (Nanjing keyGen Biotech Company, Nanjing, China). The nuclei were then counterstained with PI. After the dual staining, the cells were screened by a FAC Scan flow cytometer (FACS Calibun, Becton Dickinson, Nanjing, China). The upper left corner of the quadrant represents debris, lower left are live cells, upper right are advanced apoptotic or necrotic cells and lower right are apoptotic cells.

### 3.10. Molecular Modeling

Compounds **8h** and **8t** were prepared by the protein preparation wizard in Maestro with standard settings. Grids of CDK2 and FLT3 were generated using Glide, version 10.2, following the standard procedure recommended by Schrodinger. Then **8h** and **8t** were docked into CDK2 (PDB code: 2VU3) and FLT3 with DFG-in conformation as previous reported [22].

## 4. Conclusions

In summary, a series of 1*H*-pyrazole-3-carboxamides derivatives were designed and synthesized. The step-by-step structural optimization demonstrated that the combination of piperazine in the hydrophilic pocket, a benzene ring as aromatic linker, and a bulkier fused ring in the deep hydrophobic pocket can significantly increase the inhibitory activity of these kinds of compounds against CDK2/4 and FLT3. Among these compounds, compound **8t** showed significant potency against CDKs and FLT3, almost 10 times more powerful than FN-1501. Compound **8t** also exhibited significant inhibitory activity against various FLT3 mutations, especially against FLT3 (ITD)-F691L, indicating its potential to overcome drug resistance caused by FLT3 mutation. Compound **8t** showed potent anti-proliferative activity against a variety of cancer cell lines including MV4-11 cells, and inhibited phosphorylation of CDK and FLT3 pathways in a dose-dependent manner. These results demonstrated the potential of this compound (**8t**) for further development as a promising agent for treatment of AML as well as other cancers.

## Figures and Tables

**Table 1:** FLT3 tyrosine kinase inhibitors under clinical investigation.

FLT3 Inhibitors	Selectivity	Generation	Targets	Phases of Development (AML)	
Sunitinib (Type I)	Non-selective	First-generation	c-KIT, KDR, PDGFR, and FLT3	Phase II	
Lestaurtinib (Type I)	Non-selective	First-generation	Mutant and wild-type FLT3, JAK2, and FLT3	Phase II	
Midostaurin (Type I)	Non-selective	First-generation	FLT3, FLT3-ITD and FLT3-TKD	Launched	
Sorafenib (Type II)	Non-selective	First-generation	RAF-1, VEGFR, PDGFR, c-KIT and FLT3	Phase II	
FN-1501 (Type I)	Non-selective	First-generation	FLT3, CDKs	Phase I ^1^	
Quizartinib (Type II)	Selective	Second-generation	PDGFR, c-KIT, FLT3, CSF-1R and RET	Launched (In Japan)	
Gilteritinib (Type II)	Selective	Second-generation	FLT3 and AXL	Launched	
Crenolanib (Type II)	Selective	Second-generation	FLT3 and PDGFR α/β	Phase III	

^1^ Currently, under Phase I clinical trial for the advanced solid tumor in USA.

**Table 2:**
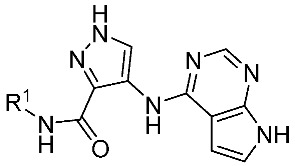
Structures and biological activities of compounds **3a**–**3d**.

Cpd.	R ^1^	IC_50_ (nM) ^1^		
		CDK2	CDK4	FLT3
**FN-1501**	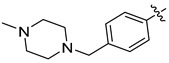	2.33 ± 0.02	1.02 ± 0.16	0.39 ± 0.07
**3a**	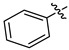	0.20 ± 0.01	34.13 ± 0.94	5.10 ± 0.46
**3b**	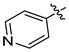	4.31 ± 0.91	54.24 ± 1.26	5.83 ± 0.74
**3c**	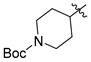	32.81 ± 1.34	87.07 ± 1.26	88.76 ± 1.06
**3d**	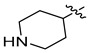	63.21 ± 0.91	77.37 ± 1.10	74.30 ± 1.21

^1^ In the presence of 10 μM ATP, the values are the mean ± SD from three independent experiments.

**Table 3:**
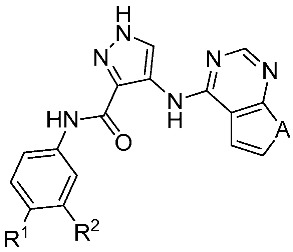
Structures and biological activities of compounds **8a**–**8j.**

Cpd.	Structure			IC_50_ (nM) ^1^			IC_50_ (nM) ^2^
	R ^1^	R ^2^	A	CDK2	CDK4	FLT3	MV4-11
**FN-1501**	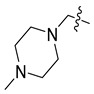	**H**	**NH**	2.33 ± 0.02	1.02 ± 0.16	0.39 ± 0.07	9 ± 0.27
**8a**	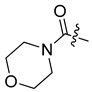	**H**	**S**	10.39 ± 0.41	32.99 ± 0.94	17.81 ± 0.89	33.10 ± 0. 17
**8b**	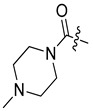	**H**	**S**	8.42 ± 0.52	30.14 ± 0.99	19.18 ± 0.18	35.21 ± 0.83
**8c**	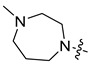	**H**	**S**	3.51 ± 0.19	2.41 ± 0.21	0.176 ± 0.09	4.28 ± 0.35
**8d**	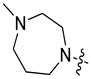	**H**	**NH**	2.32 ± 0.014	5.32 ± 0.31	0.262 ± 0.01	9.5 ± 0.01
**8e**	**H**	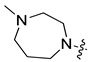	**NH**	5.49 ± 0.42	51.035 ± 0.88	2.71 ± 0.31	38.3 ± 1.21
**8f**	**H**	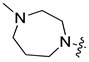	**S**	31.7 ± 0.55	67.28 ± 1.09	8.07 ± 0.21	54.15 ± 1.73
**8g**	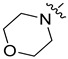	**H**	**NH**	3.74 ± 0.16	10.605 ± 0.24	1.945 ± 0.013	16.02 ± 0.43
**8h**	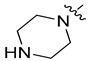	**H**	**NH**	0.282 ± 0.013	1.19 ± 0.09	0.038 ± 0.001	7.3 ± 0.33
**8i**	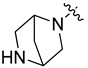	**H**	**S**	24.53 ± 0.57	9.165 ± 0.33	3.24 ± 0.14	27.21 ± 0.43
**8j**	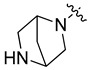	**H**	**NH**	9.64 ± 0.46	17.79 ± 0.82	2.81 ± 0.11	21.35 ± 0.56

^1^ In the presence of 10 μM ATP, the highest test concentration is 1 μM. The values are the mean ± SD from three independent experiments. ^2^ The highest test concentration is 1 μM, the values are the mean ± SD from three independent experiments.

**Table 4:**
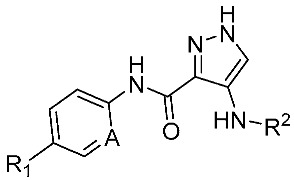
Structures and biological activities of compounds **8k**–**8t**.

Cpd.	Structure			IC_50_ (nM) ^1^			IC_50_ (nM) ^2^
	R ^1^	A	R ^2^	CDK2	CDK4	FLT3	MV4-11
**8k**	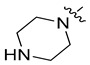	**N**	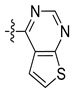	315.21 ± 2.30	22.3 ± 0.92	6.03 ± 0.16	51.09 ± 1.34
**8l**	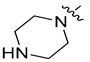	**N**	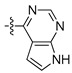	156.54 ± 3.22	23.11 ± 0.55	13.83 ± 0.37	133.50 ± 1.64
**8m**	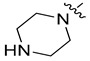	**N**	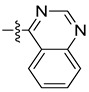	133.37 ± 1.74	17.53 ± 0.88	23.69 ± 0.65	143.50 ± 1.21
**8n**	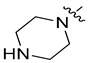	**N**	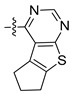	98.72 ± 1.33	4.85 ± 0.20	1.88 ± 0.09	19.92 ± 0.74
**8o**	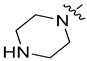	**N**	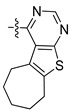	109.21 ± 1.01	1.81 ± 0.023	8.28 ± 0.12	45.44 ± 1.07
**8p**	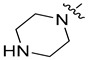	**N**	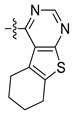	86.36 ± 1.36	3.81 ± 0.26	7.45 ± 0.19	27.04 ± 0. 31
**8q**	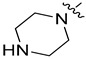	**N**	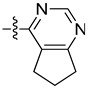	508.94 ± 10.33	123.37 ± 1.09	20.23 ± 0.35	458.32 ± 9.20
**8r**	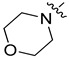	**CH**	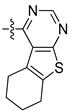	9.29 ± 0.64	24.95 ± 0.29	3.80 ± 0.10	3.28 ± 0.19
**8s**	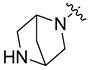	**CH**	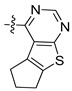	5.43 ± 0.41	4.36 ± 0.19	0.82 ± 0.003	9.13 ± 0.11
**8t**	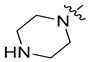	**CH**	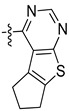	0.719 ± 0.064	0.770 ± 0.007	0.089 ± 0.013	1.22 ± 0.06

^1^ In the presence of 10 μM ATP, the highest test concentration is 1 μM. The values are the mean ± SD from three independent experiments. ^2^ The highest test concentration is 1 μM. The values are the mean ± SD from three independent experiments.

**Table 5:** Inhibition of compound **8t** against CDKs, FLT3s, and other kinases.

Kinase	IC_50_ (nM)
	Compound 8t
CDK1/cyclin B	138.03 ± 1.24
CDK2/cyclin A	2.56 ± 0.31
CDK3/cyclin E	6.88 ± 0.25
CDK4/cyclin D1	0.78 ± 0.04
CDK5/p35	9.64 ± 0.81
CDK6/cyclin D1	0.59 ± 0.09
CDK7/cyclin H	21.31 ± 1.01
CDK9/cyclin K	14.20 ± 0.82
ERK7/MAPK15	9.57 ± 0.23
FLT1/VEGFR1	12.41 ± 0.19
FLT3	0.035 ± 0.01
FLT3 (D835Y)	0.75 ± 0.04
FLT3 (F594_R595insR)	0.63 ± 0.09
FLT3 (F594_R595insREY)	1.38 ± 0.11
FLT3 (ITD)-NPOS	4.32 ± 0.23
FLT3 (ITD)-W51	0.94 ± 0.09
FLT3 (R595_E596insEY)	1.59 ± 0.14
FLT3 (Y591-V592insVDFREYEYD)	0.66 ± 0.08
FLT3 (ITD)-F691L	0.60 ± 0.01
FLT4/VEGFR3	4.16 ± 0.19
GSK3β	11.99 ± 1.20
KDR/VEGFR2	8.32 ± 0.54

**Table 6:** In vitro antiproliferative activity of compound **8t** from NCI60 screening.

Panel	Cell Line	GI_50_ (μM)	Panel	Cell Line	GI_50_ (μM)
**Leukemia**	CCRF-CEM	0.22	**Melanoma**	LOX IMVI	0.20
	HL-60(TB)	1.15		MALME-3M	0.24
	K-562	0.12		M14	0.12
	MOLT-4	0.08		MDA-MB-435	0.11
	RPMI-8226	0.89		SK-MEL-2	1.16
	SR	0.06		SK-MEL-5	0.25
**Non-Small Cell Lung Cancer**	A549/ATCC	0.14		SK-MEL-28	1.13
	EKVX	0.06		UACC-257	0.48
	HOP-62	0.37		UACC-62	1.09
	HOP-92	0.04	**Ovarian Cancer**	IGROV1	0.12
	NCI-H226	0.32		OVCAR-3	0.23
	NCI-H23	0.26		OVCAR-4	0.22
	NCI-H322M	0.14		OVCAR-5	0.27
	NCI-H460	0.06		OVCAR-8	0.36
	NCI-H522	0.21		NCI/ADR-RES	1.48
**Colon Cancer**	COLO 205	0.12		SK-OV-3	0.26
	HCC-2998	1.20	**Renal Cancer**	786-0	0.36
	HCT-116	0.11		A498	0.14
	HCT-15	0.22		ACHN	0.16
	HT29	0.26		CAKI-1	0.08
	SW-620	0.12		RXF 393	0.39
	KM12	0.17		SN12C	0.45
**CNS Cancer**	SF-268	0.72		TK-10	0.47
	SF-295	0.27		UO-31	0.40
	SF-539	0.39	**Breast Cancer**	MCF7	0.16
	SNB-19	0.24		MDA-MB-231	1.93
	SNB-75	0.05		HS 578T	0.10
	U251	0.11		BT-549	1.02
**Prostate Cancer**	PC-3	0.18		T-47D	0.59
	DU-145	0.30		MDA-MB-468	0.42

## Supplementary Materials

Supplementary materials can be found at https://www.mdpi.com/1422-0067/20/22/5739/s1.

## Abbreviations

FLT3	Fms-like receptor tyrosine kinase 3
AML	acute myeloid leukemia
ALL	acute lymphoblastic leukemia
ITD	internal-tandem duplication
TKD	tyrosine kinase domain
CDK	cyclin-dependent kinases
