# Supplementary material for: Design and Synthesis of 4-(Heterocyclic Substituted Amino)-1H-Pyrazole-3-Carboxamide Derivatives and Their Potent Activity against Acute Myeloid Leukemia (AML)
Source: Int J Mol Sci. 2019 Nov 15;20(22):5739. doi: 10.3390/ijms20225739 (PMC6887723; doi:10.3390/ijms20225739)
Supplement: Supplementary file 1 [file ijms-20-05739-s001.pdf]

International Journal of
Molecular Sciences

Supplemental information

Design and Synthesis of 4-(Heterocyclic Substituted Amino)-1*H*-Pyrazole-3-Carboxamide Derivatives and Their Potent Activity against Acute Myeloid Leukemia (AML)

Yanle Zhi [1,2,3], Zhijie Wang [4], Chao Yao [4], Baoquan Li [4], Hao Heng [4], Jiongheng Cai [4], Li Xiang [2], Yue Wang [4], Tao Lu [4,*] and Shuai Lu [4,*]

[1] School of Pharmacy, Henan University of Chinese Medicine, Zhengzhou 450046, China; 15311050167@stu.cpu.edu.cn
[2] School of Pharmacy, China Pharmaceutical University, 24 Tongjiaxiang, Nanjing 210009, China; 16401607@stu.cpu.edu.cn
[3] Collaborative Innovation Center for Respiratory Disease Diagnosis, Treatment & Chinese Medicine Development of Henan Province, Henan University of Chinese Medicine, Zhengzhou, 450046, China
[4] School of Science, China Pharmaceutical University, 639 Longmian Avenue, Nanjing 211198, China; 3119050171@stu.cpu.edu.cn (Z.W.); 15211050685@stu.cpu.edu.cn (C.Y.); 15221051005@stu.cpu.edu.cn (B.L.); dg1924026@smail.nju.edu.cn (H.H.); 3319051009@stu.cpu.edu.cn (J.C.); 1020001155@cpu.edu.cn (Y.W.)
* Correspondence: lu_shuai@cpu.edu.cn (S.L.); lutao@cpu.edu.cn (T.L.); Tel: +86-25-86185153 (S.L.); +86-25-83271555 (T.L.)

Table S1. Enzyme activity of compound **8t** against the selected kinases.

**Kinase**	**% Enzyme Activity (Relative to DMSO Controls) 0.15 μM**
AKT1	122.27
ABL1	81.04
ALK	16.58
ALK1/ACVR1	57.96
Aurora A	38.00
Aurora B	55.33
BRAF	77.62
BTK	97.07
CDK1/cyclin B	18.21
CDK2/cyclin A	4.01
CDK3/cyclin E	5.94
CDK4/cyclin D1	1.56
CDK5/p35	9.60
CDK6/cyclin D1	1.33
CDK7/cyclin H	19.90
CDK9/cyclin K	7.20
CHK2	38.38
c-Kit	64.79
c-MET	98.97
c-Src	73.76
ERK7/MAPK15	2.96
FGFR1	40.05
FLT1/VEGFR1	6.08
FLT3	0.01
FLT3 (D835Y)	0.27
FLT3 (F594_R595insR)	0.47
FLT3 (F594_R595insREY)	0.80
FLT3 (ITD)-NPOS	1.38
FLT3 (ITD)-W51	0.51
FLT3 (R595_E596insEY)	0.44
FLT3 (Y591-V592insVDFREYEYD)	0.54

FLT4/VEGFR3	3.62
FMS	63.04
GSK3b	8.65
JAK2	32.02
JNK1	39.22
KDR/VEGFR2	7.29
PDGFRa	82.79
SYK	35.40

Table S2. IC_{50} of compound **8t**, Quizartinib and FN-1501 against BaF3 (FLT3 (ITD-F691L)).

**Compound**	**IC_{50} (nM)** **BaF3 (FLT3 (ITD-F691L))**
**8t**	21.9
Quizartinib	70.8
FN-1501	55.9

Synthesis of intermediates

*Procedure A For the Synthesis of Compounds **4a** and **4b***

The mixture of appropriate amine (18.5 mmol), p-nitrobenzoic acid (20.4 mmol), EDC (22.2 mmol), HOBT (22.2 mmol) in DMF (30 mL) was stirred for 24 hours. The ice water (100 mL) was added to the reaction mixture. A large amount of yellow solid precipitation (compounds **4a** and **4b**) was acquired. Compounds **4a** and **4b** were used without further purification.

*Procedure B For the Synthesis of Compounds **4c–4h***

Fluorobenzene or fluoropyridine (46.3 mmol) and K_2CO_3 (69.5 mmol) were dissolved in DMSO (50 mL). The reaction mixture was stirred at r.t. for 30 min and then amine (69.5 mmol) was added. The reaction mixture was stirred at 70 °C for 5 h. The ice water (500 mL) was added to the reaction mixture. A large amount of yellow solid precipitation (compounds **4c–4h**) was acquired. Compounds **4c–4h** were used for further reaction without purification.

*Procedure C For the Synthesis of Compounds **2a–2c**, **5a–5h** and **7a–7h***

To a suspension of compounds **1a–1c**, **4a–4h**, **6c–6h** or **4a–4h** (26.2 mmol) in 95% ethanol (100 mL), 85% $NH_2NH_2{\cdot}H2O$ (262 mmol), 95% ethanol (100 mL) and iron (III) oxide hydroxide (FeO(OH)/C, 0.5 g) were added and heated to reflux. When TLC analysis showed complete conversion of the starting material, the reaction mixture was filtrate through Celite® and the filtrate was concentrated in vacuum. The crude product was purified by silica gel column chromatography (DCM/MeOH) to yield the title compound as white solid.

*Procedure D for the synthesis of compounds **1a–1c** and **6a–6h***

4-nitro-1*H*-pyrazole-3-carboxylic acid (4.19 g, 13.94 mmol) was dissolved in 20 mL THF, DMF (0.5 mL) and oxalyl chloride (1.78 mL, 20.91 mmol) were added at 0 °C, the resultant mixture was stirred at room temperature for 60 min. After the mixture was concentrated in vacuo, the residue was dissolved in pyridine (20 mL), and the solution was added dropwise into the solution of 7 (dissolved in 20 mL pyridine) at 0 °C. The solution was stirred for 6 h at 25 °C. Upon completion of the reaction, the solvent was removed on a rotary evaporator. Then water (100 mL) was added, and the mixture was basified using 10% NaOH until pH 8~9. The solid precipitation was filtered to give the crude product, which was used for next step without further purification.

Procedure E For the Synthesis of Compounds 3a–3c, 8a–8g and 8r

Compounds **2a–2c**, **7a–7b** or **7e–7h** (10 mmol) were reacted with corresponding chlorides (12 mmol) in AcOH/H_2O:1/1 (10 mL) at 50 °C. When TLC analysis showed complete conversion of the starting material, 10% NaOH was added and the pH was adjust to 8–9. The precipitate was collected

and purified by silica gel column chromatography with DCM/MeOH (30/1) to yield the compound **3a–3c**, **8a–8g** and **8r**.

Procedure F For the Synthesis of Compounds 3d, 8h–8q and 8s–8t

Compounds **3c**, **7c–7d** or **7g** (10 mmol) were reacted with corresponding chlorides (12 mmol) in AcOH/H2O:1/1 (10 mL) at 50 °C. When TLC analysis showed complete conversion of the **2c**, **7c–7d** or **7g,** increasng the reaction temperature to 70 °C (5 h) to the cleavage of *t*-butylcarbamoyl group. Upon completion of the reaction, 10% NaOH was added and the pH was adjusted to 8–9. The precipitate was collected and purified by silica gel column chromatography with DCM/MeOH (30/1) to yield the compounds **3d**, **8h–8q** and **8s–8t**.

(4-Aminophenyl)(morpholino)methanone (**5a**). Compound **5a** was prepared according to procedure C on 10 mmol scale. Purification by column chromatography (2.5% MeOH/DCM) yielded the title compound (1.92 g, 9.3 mmol, Yield 93%). $[M+H]^+$: 207.11.

(4-Aminophenyl)(4-methylpiperazin-1-yl)methanone (**5b**). Compound **5b** was prepared according to procedure C on 10 mmol scale. Purification by column chromatography (2.5% MeOH/DCM) yielded the title compound (2.03 g, 9.2 mmol, Yield 92%). $[M+H]^+$: 220.14.

***tert*-Butyl-4-(6-aminopyridin-3-yl)piperazine-1-carboxylate** (**5c**). Compound **5c** was prepared according to procedure C on 10 mmol scale. Purification by column chromatography (5% MeOH/DCM) yielded the title compound (2.6 g, 9.2 mmol, Yield 92%). $[M+H]^+$: 279.18.

***tert*-Butyl-4-(4-aminophenyl)piperazine-1-carboxylate** (**5d**). Compound **5d** was prepared according to procedure C on 10 mmol scale. Purification by column chromatography (5% MeOH/DCM) yielded the title compound (2.7 g, 9.6 mmol, Yield 96%). $[M+H]^+$: 278.18.

4-Morpholinoaniline (**5e**). Compound **5e** was prepared according to procedure C on 10 mmol scale. Purification by column chromatography (5% MeOH/DCM) yielded the title compound (1.7 g, 9.6 mmol, Yield 96%). $[M+H]^+$: 179.11.

4-(4-Methyl-1,4-diazepan-1-yl)aniline (**5f**). Compound **5f** was prepared according to procedure C on 10 mmol scale. Purification by column chromatography (2.5% MeOH/DCM) yielded the title compound (1.68 g, 8.2 mmol, Yield 82%). $[M+H]^+$: 206.16.

***tert*-Butyl-5-(4-aminophenyl)-2,5-diazabicyclo[2.2.1]heptane-2-carboxylate** (**5g**). Compound **5g** was prepared according to procedure C on 10 mmol scale. Purification by column chromatography (2.5% MeOH/DCM) yielded the title compound (2.11 g, 7.3 mmol, Yield 73%). $[M+H]^+$: 290.18.

3-(4-Methyl-1,4-diazepan-1-yl)aniline (**5h**). Compound **5h** was prepared according to procedure C on 10 mmol scale. Purification by column chromatography (2.5% MeOH/DCM) yielded the title compound (2.11 g, 7.3 mmol, Yield 73%). $[M+H]^+$: 290.18.

4-Amino-*N*-phenyl-1*H*-pyrazole-3-carboxamide (2a). Compound **2a** was prepared according to procedure C on 5 mmol scale. Purification by column chromatography (5% MeOH/DCM) yielded the title compound (0.77 g, 3.8 mmol, Yield 76%). $[M+H]^+$: 203.09.

4-Amino-*N*-(pyridin-4-yl)-1*H*-pyrazole-3-carboxamide (**2b**). Compound **2b** was prepared according to procedure C on 5 mmol scale. Purification by column chromatography (5% MeOH/DCM) yield the title compound (0.48 g, 2.4 mmol, Yield 47%). $[M+H]^+$: 204.09.

***tert*-Butyl-4-(4-amino-1*H*-pyrazole-3-carboxamido)piperidine-1-carboxylate** (**2c**) Compound **2c** was prepared according to procedure C on 5 mmol scale. Purification by column chromatography (5% MeOH/DCM) yielded the title compound (1.21 g, 3.9 mmol, Yield 77%). $[M+H]^+$: 310.19.

4-Amino-*N*-(4-(morpholine-4-carbonyl)phenyl)-1*H*-pyrazole-3-carboxamide (**7a**). Compound **7a** was prepared according to procedure C on 5 mmol scale. Purification by column chromatography (5% MeOH/DCM) yielded the title compound (1.10 g, 3.4 mmol, Yield 67%). $[M+H]^+$: 316.14.

4-Amino-*N*-(4-(4-methylpiperazine-1-carbonyl)phenyl)-1*H*-pyrazole-3-carboxamide (**7b**). Compound **7b** was prepared according to procedure C on 5 mmol scale. Purification by column chromatography (5% MeOH/DCM) yielded the title compound (0.75 g, 2.3 mmol, Yield 70%). $[M+H]^+$: 329.17.

tert-butyl-4-(6-(4-amino-1*H*-pyrazole-3-carboxamido)pyridin-3-yl)piperazine-1-carboxylate (**7c**) Compound **7c** was prepared similar to procedure C on 10 mmol scale. The difference is taken

anhydrous pyridine as solvent when intermediate **5c** reaction with 4-nitro-1*H*-pyrazole-3-carbonyl chloride. The crude product was purified by silica gel column chromatography with DCM/MeOH (30/1) to yield the compound **7c** (white solid, 1.0 g, 2.6 mmol Yield 52%). $[M+H]^+$: 388.20.

***tert*-Butyl-4-(4-(4-amino-1*H*-pyrazole-3-carboxamido)phenyl)piperazine-1-carboxylate** (**7d**).Compound **7d** was prepared according to procedure C on 5 mmol scale. Purification by column chromatography (5% MeOH/DCM) yielded the title compound (1.20 g, 3.2 mmol, Yield 63%). $[M+H]^+$: 387.21.

4-Amino-*N*-(4-morpholinophenyl)-1*H*-pyrazole-3-carboxamide (**7e**). Compound **7e** was prepared according to procedure C on 5 mmol scale. Purification by column chromatography (5% MeOH/DCM) yielded the title compound (1.03 g, 3.6 mmol, Yield 72%). $[M+H]^+$: 288.15.

4-Amino-*N*-(4-(4-methyl-1,4-diazepan-1-yl)phenyl)-1*H*-pyrazole-3-carboxamide (**7f**). Compound **7f** was prepared according to procedure C on 5 mmol scale. Purification by column chromatography (5% MeOH/DCM) yielded the title compound (0.69 g, 2.2 mmol, Yield 43%). $[M+H]^+$: 315.19.

***tert*-Butyl-5-(4-(4-amino-1*H*-pyrazole-3-carboxamido)phenyl)-2,5-diazabicyclo[2.2.1]heptane-2-carboxylate** (**7g**). Compound **7g** was prepared according to procedure C on 5 mmol scale. Purification by column chromatography (5% MeOH/DCM) yielded the title compound (0.92 g, 2.3 mmol, Yield 45%). $[M+H]^+$: 399.21.

4-Amino-*N*-(3-(4-methyl-1,4-diazepan-1-yl)phenyl)-1*H*-pyrazole-3-carboxamide (**7h**). Compound **7h** was prepared according to procedure C on 5 mmol scale. Purification by column chromatography (5% MeOH/DCM) yielded the title compound (0.50 g, 1.6 mmol, Yield 31%). $[M+H]^+$: 315.19.

Structural characterization

4-((7*H*-Pyrrolo[2,3-d]pyrimidin-4-yl)amino)-*N*-phenyl-1*H*-pyrazole-3-carboxamide (**3a**). Compound **3a** was prepared according to procedure G on 1 mmol scale. Purification by column chromatography (5% MeOH/DCM) yield the title compound (255 mg, 0.18 mmol, Yield 80%). White solid; m.p >300 °C. HPLC analysis: retention time = 5.248 min; peak area, 98.56%. ^{1}H NMR (300 MHz, DMSO-*d*$_6$) δ 13.44 (s, 1H, -NH-, pyrazole), 11.85 (s, 1H, -NH-, pyrrole), 10.20 (s, 1H, -NHCO-), 9.50 (s, 1H,-NH-), 8.57 (s, 1H, ArH), 8.38 (s, 1H, ArH), 7.86 (d, *J* = 6.0 Hz, 2H, ArH), 7.33 (d, *J* = 17.1 Hz, 3H, ArH), 7.12 (s, 1H, ArH), 6.49 (s, 1H, ArH); ^{13}C NMR (151 MHz, DMSO-*d*$_6$) δ 163.34 (s), 152.14 (s), 151.66 (s), 151.03 (s), 138.77 (s), 132.72 (s), 129.05 (s), 125.09 (s), 124.23 (s), 123.51 (s), 121.21 (s), 120.59 (s), 103.56 (s), 97.21 (s); HRMS-ESI m/z $[M+H]^+$ calcd for $C_{16}H_{14}N_7O$: 320.1260, found: 320.1219.

4-((7*H*-Pyrrolo[2,3-d]pyrimidin-4-yl)amino)-*N*-(pyridin-4-yl)-1*H*-pyrazole-3-carboxamide (**3b**). Compound **3b** was prepared according to procedure G on 1.0 mmol scale. Purification by column chromatography (5% MeOH/DCM) yield the title compound (190 mg, 0.59 mmol, Yield 59%). white solid; m.p >300 °C. HPLC analysis: retention time = 4.785 min; peak area, 99.32%.^{1}H NMR (300 MHz, DMSO-*d*$_6$) δ 13.62 (s, 1H, -NH-, pyrazole), 11.88 (s, 1H, -NH-, pyrrole), 10.72 (s, 1H, -NHCO-), 9.38 (s, 1H, -NH-), 8.58 (s, 1H, ArH), 8.50 (d, *J* = 2.7 Hz, 2H, ArH), 8.38 (s, 1H, ArH), 7.96 (d, *J* = 6.1 Hz, 2H, ArH), 7.31 (s, 1H, ArH), 6.52 (s, 1H, ArH); HRMS-ESI m/z $[M+H]^+$ calcd for $C_{15}H_{12}N_8O$: 320.1134, found: 320.1149.

***tert*-Butyl-4-(4-((7H-pyrrolo[2,3-d]pyrimidin-4-yl)amino)-1*H*-pyrazole-3-carboxamido)piperidine-1-carboxylate** (**3c**). Compound **3c** was prepared according to procedure G on 0.5 mmol scale. Purification by column chromatography (5% MeOH/DCM) yield the title compound (151 mg, 0.71 mmol, Yield 71%). yellow solid; m.p 268–269 °C. HPLC analysis: retention time = 6.356 min; peak area, 98.26%. ^{1}H NMR (300 MHz, DMSO-*d*$_6$) δ 13.18 (s, 1H, -NH-, pyrazole), 11.81 (s, 1H, -NH-, pyrrole), 9.68 (s, 1H, -NH-), 8.49 (s, 1H, ArH), 8.36 (s, 1H, ArH), 8.26 (d, *J* = 8.4 Hz, 1H, ArH),7.28(m, 1H, ArH), 6.42 (dd, *J* = 3.2, 1.5 Hz, 1H, ArH), 4.00 (m, 3H, -CH$_2$-， -CH-, piperidine), 2.89-2.73 (m, 2H, -CH$_2$-, piperidine), 1.76 (d, *J* = 10.3 Hz, 2H, -CH$_2$-, piperidine), 1.59-1.49 (m, 2H, -CH$_2$-, piperidine), 1.42 (s, 9H, -CH$_3$×3); ^{13}C NMR (151 MHz, DMSO-*d*$_6$) δ 163.90 (s), 154.38 (s), 152.03 (s), 151.71 (s), 150.95 (s), 132.44 (s), 124.70 (s), 123.44 (s), 119.96 (s), 103.48 (s), 97.01 (s), 79.12 (s), 46.12 (s), 43.57 (s) 31.67 (s), 28.56 (s); HRMS-ESI m/z $[M+H]^+$ calcd for $C_{20}H_{27}N_8O_3$: 427.2206, found: 427.2190.

4-((7*H*-Pyrrolo[2,3-d]pyrimidin-4-yl)amino)-*N*-(piperidin-4-yl)-1*H*-pyrazole-3-carboxamide (3d). Compound **3d** was prepared according to procedure G on 0.5 mmol scale. Purification by column chromatography (5% MeOH/DCM) yield the title compound (82 mg, 0.25 mmol, Yield 49%). Yellow solid; m.p 266–268 °C. HPLC analysis: retention time = 5.789 min; peak area, 97.34%. ^{1}H NMR (300 MHz, DMSO-d_6) δ 11.77 (s, 1H, -NH-, pyrrole), 9.64 (s, 1H, -NH-), 8.47 (s, 1H, ArH), 8.35 (s, 1H, ArH), 8.05 (d, *J* = 7.8 Hz, 1H, ArH), 7.27 (d, *J* = 2.8 Hz, 1H, ArH), 6.43 (d, *J* = 3.0 Hz, 1H, ArH), 3.92 (s, 1H, -CH-, piperidine), 3.00 (d, *J* = 8.1 Hz, 2H, -CH$_2$-, piperidine), 2.58 (s, 2H, -CH$_2$-, piperidine), 1.76 (d, *J* = 10.4 Hz, 2H, -CH$_2$-, piperidine), 1.53 (d, *J* = 9.3 Hz, 2H, -CH$_2$-, piperidine), 0.84 (d, *J* = 5.8 Hz, 1H, -NH-, piperidine); ^{13}C NMR (151 MHz, DMSO-d_6) δ 163.56 (s), 152.10 (s), 151.71 (s), 150.93 (s), 131.88 (s), 124.77 (s), 123.44 (s), 120.86 (s), 103.48 (s), 97.07 (s), 46.42 (s), 45.12 (s), 29.51 (s); HRMS-ESI m/z [M+H]$^+$ calcd for $C_{15}H_{19}N_8O$: 327.1682, found: 327.1646.

***N*-(4-(Morpholine-4-carbonyl)phenyl)-4-(thieno[2,3-d]pyrimidin-4-ylamino)-1*H*-pyrazole-3-carboxamide (8a)**. Compound **8a** was prepared according to procedure G on 0.5 mmol scale. Purification by column chromatography (5% MeOH/DCM) yield the title compound (122 mg, 0.27 mmol, Yield 54 %). White solid; m.p 243–244 °C. HPLC analysis: retention time = 3.991 min; peak area, 98.23%. ^{1}H NMR (300 MHz, DMSO-d_6) δ 10.60 (s, 1H, -NHCO-), 10.46 (s, 1H, -NH-), 8.67 (s, 1H, ArH, pyrazole), 8.48 (s, 1H, ArH, Pyrimidine), 7.93 (d, *J* = 8.7 Hz, 2H, ArH), 7.88 (d, *J* = 5.9 Hz, 1H, ArH, Thiophene), 7.69 (d, *J* = 6.1 Hz, 1H, ArH, Thiophene), 7.41 (d, *J* = 8.7 Hz, 2H, ArH), 3.61 (s, 4H, -CH$_2$-×2), 3.51 (s, 4H, -CH$_2$-×2); ^{13}C NMR (151 MHz, DMSO-d_6) δ 169.34 (s), 161.85 (s), 153.80 (s), 150.43 (s), 140.11 (s), 135.76 (s), 130.93 (s), 128.36 (s), 127.07 (s), 125.66 (s), 121.18 (s), 120.47 (s), 119.44 (s), 117.55 (s), 66.57 (s), 48.16 (s); HRMS-ESI m/z [M+H]$^+$ calcd for $C_{21}H_{20}N_7O_3S$: 450.1348, found: 450.1303.

***N*-(4-(4-Methylpiperazine-1-carbonyl)phenyl)-4-(thieno[2,3-d]pyrimidin-4-ylamino)-1*H*-pyrazole-3-carboxamide (8b)**. Compound **8b** was prepared according to procedure G on 0.5 mmol scale. Purification by column chromatography (5% MeOH/DCM) yield the title compound (134 mg, 0.29 mmol, Yield 58 %). White solid; m.p 280–281 °C. HPLC analysis: retention time = 4.102 min; peak area, 98.43%. ^{1}H NMR (300 MHz, DMSO-d_6) δ 13.55 (s, 1H, -NH-, pyrazole), 10.54 (s, 1H, -NHCO-), 9.94 (s, 1H, -NH-), 8.61 (s, 1H, ArH, pyrazole), 8.56 (s, 1H, ArH, Pyrimidine), 7.96 (s, 1H, ArH), 7.93 (s, 1H, ArH), 7.80 (d, *J* = 6.0 Hz, 1H, ArH), 7.52 (d, *J* = 6.0 Hz, 1H, ArH), 7.41 (d, *J* = 1.9 Hz, 1H, ArH), 7.39 (d, *J* = 1.9 Hz, 1H, ArH), 3.49 (s, 4H, -CH$_2$-×2), 2.32 (s, 4H, -CH$_2$-×2), 2.20 (s, 3H, -CH$_3$); ^{13}C NMR (151 MHz, DMSO-d_6) δ 169.25 (s), 166.46 (s), 154.05 (s), 153.51 (s), 139.94 (s), 133.46 (s), 131.39 (s), 128.21 (s), 125.51 (s), 124.03 (s), 122.09 (s), 120.65 (s), 118.13 (s), 117.01 (s), 55.00 (s), 47.65 (s), 46.09 (s); HRMS-ESI m/z [M+H]$^+$ calcd for $C_{22}H_{23}N_8O_2S$: 463.1665, found: 463.1632.

***N*-(4-(4-Methyl-1,4-diazepan-1-yl)phenyl)-4-(thieno[2,3-d]pyrimidin-4-ylamino)-1*H*-pyrazole-3-carboxamide (8c)**. Compound **8c** was prepared according to procedure G on 0.6 mmol scale. Purification by column chromatography (5% MeOH/DCM) yield the title compound (108 mg, 0.24 mmol, Yield 48%). white solid; m.p 223–224 °C. HPLC analysis: retention time = 4.657 min; peak area, 97.84%.^{1}H NMR (300 MHz, DMSO-d_6) δ 13.48 (s, 1H, -NH-, pyrazole), 10.14 (s, 1H, -NHCO-), 10.04 (s, 1H, -NH-), 8.61 (s, 1H, ArH, pyrazole), 8.53 (s, 1H, ArH, Pyrimidine), 7.79 (d, *J* = 6.0 Hz, 1H, ArH, Thiophene), 7.59 (d, *J* = 8.9 Hz, 2H, ArH), 7.47 (d, *J* = 6.0 Hz, 1H, ArH, Thiophene), 6.68 (d, *J* = 9.0 Hz, 2H, ArH), 3.57 – 3.48 (m, 2H, -CH$_2$-, Homopiperazine), 3.43 (t, *J* = 6.2 Hz, 2H, -CH$_2$-, Homopiperazine), 2.66 (s, 2H -CH$_2$-, Homopiperazine), 2.51 (s, 2H, -CH$_2$-, Homopiperazine), 2.30 (s, 3H, -CH$_3$), 1.91 (m, 2H, -CH$_2$-, homopiperazine); ^{13}C NMR (151 MHz, DMSO-d_6) δ 166.36 (s), 162.30 (s), 154.10 (s), 153.33 (s), 146.16 (s), 133.38 (s), 127.15 (s), 125.52 (s), 123.77 (s), 123.13 (s), 117.98 (s), 116.95 (s), 111.56 (s), 57.49 (s), 56.73 (s), 48.33 (s), 48.21 (s), 46.33 (s), 27.15 (s); HRMS-ESI m/z [M+H]$^+$ calcd for $C_{22}H_{25}N_8OS$: 449.1872, found: 449.1828.

4-((7*H*-Pyrrolo[2,3-d]pyrimidin-4-yl)amino)-*N*-(4-(4-methyl-1,4-diazepan-1-yl)phenyl)-1*H*-pyrazole-3-carboxamide (8d). Compound **8d** was prepared according to procedure G on 0.5 mmol scale. Purification by column chromatography (5% MeOH/DCM) yield the title compound (144 mg, 0.32 mmol, Yield 64%). White solid; m.p 263–264 °C. HPLC analysis: retention time = 6.368 min; peak area, 98.44%.^{1}H-NMR (300 MHz, DMSO-d_6) δ 13.51 (s, 1H, -NH-, pyrazole), 10.37 (s, 1H, -NHCO-), 9.93 (s, 1H, -NH-), 8.59 (s, 1H, ArH, pyrazole), 7.80 (d, *J* = 8.3 Hz, 2H, ArH), 7.68 (d, *J* = 5.9 Hz, 1H, ArH), 7.41 (d, *J* = 6.0 Hz, 1H, ArH), 7.28 (d, *J* = 8.4 Hz, 2H, ArH), 3.58 (s, 4H, -CH$_2$-×2), 3.43 (s, 2H, -

CH_2-), 2.61 (s, 3H, -CH_3), 2.35 (s, 4H, -CH_2-×2); ^{13}C-NMR (151 MHz, DMSO-d_6) δ 162.53 (s), 151.71 (s), 123.47 (s), 123.12 (s), 111.63 (s), 97.16 (s), 57.45 (s), 56.67 (s), 48.17 (s), 46.19 (s), 26.99 (s); HRMS-ESI m/z $[M+H]^+$ calcd for $C_{22}H_{24}N_7O_2S$: 432.2260, found: 432.2258.

4-((7*H*-Pyrrolo[2,3-d]pyrimidin-4-yl)amino)-*N*-(3-(4-methyl-1,4-diazepan-1-yl)phenyl)-1*H*-pyrazole-3-carboxamide (**8e**). Compound **8e** was prepared according to procedure G on 0.5 mmol scale. Purification by column chromatography (5% MeOH/DCM) yield the title compound (82 mg, 0.19 mmol, Yield 38 %). White solid; m.p 215–216 °C. HPLC analysis: retention time = 3.594 min; peak area, 99.71%. ^{1}H NMR (300 MHz, DMSO-d_6) δ 13.49 (s, 1H, -NH-, pyrazole), 11.85 (s, 1H, -NH-, pyrrole), 10.09 (s, 1H, -NHCO-), 9.90 (s, 1H, -NH-), 8.70(s, 1H, ArH, pyrazole), 8.47 (s, 1H, ArH, Pyrimidine), 7.70 (d, *J* = 6.1 Hz, 2H, ArH), 7.52 (d, *J* = 6.1 Hz, 2H, ArH), 7.31 (d, *J* = 7.7 Hz, 1H, ArH, pyrrole), 7.02-7.19 (m, 1H, ArH, pyrrole), 3.46 (s, 2H, -CH_2-), 3.40-3.44 (m, 2H, -CH_2-), 2.69 (s, 2H, -CH_2-), 2.31 (s, 3H, , -CH_3), 1.89 (s, 2H, -CH_2-), 1.10-1.12 (m, 2H, -CH_2-); ^{13}C NMR (151 MHz, DMSO-d_6) δ 163.74 (s), 151.21 (s), 151.19 (s), 150.99 (s), 146.54 (s), 129.31 (s), 124.99 (s), 123.57 (s), 122.61 (s), 111.66 (s), 105.31 (s), 97.29 (s), 59.37 (s), 57.49 (s), 47.37 (s), 45.22 (s), 27.56 (s); HRMS-ESI m/z $[M+H]^+$ calcd for $C_{22}H_{26}N_9O$: 432.2260, found: 432.2268.

***N*-(3-(4-Methyl-1,4-diazepan-1-yl)phenyl)-4-(thieno[2,3-d]pyrimidin-4-ylamino)-1*H*-pyrazole-3-carboxamide** (**8f**). Compound **8f** was prepared according to procedure G on 0.5 mmol scale. Purification by column chromatography (5% MeOH/DCM) yield the title compound (90 mg, 0.20 mmol, Yield 39 %). White solid; m.p > 300 °C. HPLC analysis: retention time = 5.354 min; peak area, 99.73%.^{1}H NMR (300 MHz, DMSO-d_6) δ 13.54 (s, 1H, -NH-, pyrazole), 10.05 (s, 1H, -NHCO-), 9.99 (s, 1H, -NH-), 8.66 (s, 1H, ArH, pyrazole), 8.54 (s, 1H, ArH, Pyrimidine), 7.80 (d, *J* = 6.0 Hz, 2H, ArH), 7.5.0 (d, *J* = 6 Hz, 2H, ArH), 7.27 (d, *J* = 7.7 Hz, 1H, ArH, Thiophene), 7.08-7.16 (m, 1H, ArH, Thiophene), 3.53 (s, 2H, -CH_2-)， 3.41-3.45 (m, 2H, -CH_2-), 2.73 (s, 2H, -CH_2-), 2.35 (s, 3H, -CH_3), 1.99 (s, 2H, -CH_2-), 1.11-1.13 (m, 2H, -CH_2-); ^{13}C NMR (151 MHz, DMSO-d_6) δ 166.21 (s), 161.30 (s), 156.22 (s), 151.35 (s), 144.31 (s), 132.18 (s), 127.35 (s), 125.22 (s), 123.71 (s), 123.14 (s), 118.91 (s), 115.34 (s), 110.62 (s), 59.31 (s), 57.65 (s), 48.22 (s), 45.34 (s), 28.11 (s); HRMS-ESI m/z $[M+H]^+$ calcd for $C_{22}H_{25}N_8OS$: 449.1872, found: 449.1876.

4-((7*H*-Pyrrolo[2,3-d]pyrimidin-4-yl)amino)-*N*-(4-morpholinophenyl)-1*H*-pyrazole-3-carboxamide (**8g**). Compound **8g** was prepared according to procedure G on 0.5 mmol scale. Purification by column chromatography (5% MeOH/DCM) yield the title compound (99 mg, 0.25 mmol, Yield 49%). Yellow solid; m.p 303–304 °C. HPLC analysis: retention time = 4.012 min; peak area, 98.89%. ^{1}H NMR (300MHz, DMSO-d_6) δ 13.38 (s, 1H, -NH-, pyrazole), 11.88 (s, 1H, -NH-, pyrrole), 10.10 (s, 1H, -NHCO-), 9.59 (s, 1H, -NH-), 8.56 (s, 1H, ArH, pyrazole), 8.39 (s, 1H, ArH, Pyrimidine), 7.72 (s, 1H, ArH), 7.69 (s, 1H, ArH), 7.31 (d, *J* = 3.4 Hz, 1H, ArH), 6.96 (s, 1H, ArH), 6.93 (s, 1H, ArH), 6.48 (d, *J* = 3.5 Hz, 1H, ArH), 3.76 (d, *J* = 4.5 Hz, 4H, -CH_2-×2), 3.08 (m, 4H, -CH_2-×2); ^{13}C NMR (151 MHz, DMSO-d_6) δ 162.84 (s), 152.13 (s), 151.69 (s), 151.02 (s), 148.13 (s), 132.78 (s), 130.83 (s), 124.97 (s), 123.49 (s), 122.36 (s), 120.45 (s), 115.70 (s), 103.54 (s), 97.20 (s), 66.58 (s), 49.32 (s); HRMS-ESI m/z $[M+H]^+$ calcd for $C_{20}H_{21}N_8O_2$: 405.1787, found: 405.1752.

4-((7*H*-Pyrrolo[2,3-d]pyrimidin-4-yl)amino)-*N*-(4-(piperazin-1-yl)phenyl)-1*H*-pyrazole-3-carboxamide (**8h**). Compound **8h** was prepared according to procedure F on 0.5 mmol scale. Purification by column chromatography (5% MeOH/DCM) yield the title compound (89 mg, 0.22 mmol, Yield 44%). White solid; m.p 304–305 °C. HPLC analysis: retention time = 4.341 min; peak area, 99.71%. ^{1}H NMR (300 MHz, DMSO-d_6) δ 13.44 (s, 1H, -NH-, pyrazole), 11.90 (s, 1H, -NH-, pyrrole), 10.11 (s, 1H, -NHCO-), 9.58 (s, 1H, -NH-), 8.56 (s, 1H, ArH, pyrazole), 8.39 (s, 1H, ArH, Pyrimidine), 7.70 (d, *J* = 9.1 Hz, 2H, ArH), 7.31 (d, *J* = 3.3 Hz, 1H, ArH, pyrrole), 6.96 (d, *J* = 9.1 Hz, 2H, ArH), 6.48 (d, *J* = 3.3 Hz, 1H, ArH, pyrrole), 3.15 (dd, J = 6.4, 3.4 Hz, 4H, -CH_2-×2), 3.03 (dd, *J* = 6.1, 3.5 Hz, 4H, -CH_2-×2); ^{13}C NMR (151 MHz, DMSO-d_6) δ 162.79 (s), 152.14 (s), 151.70 (s), 151.02 (s), 147.98 (s), 132.54 (s), 130.97 (s), 124.98 (s), 123.50 (s), 122.38 (s), 120.90 (s), 116.30 (s), 103.54 (s), 97.18 (s), 48.51 (s), 44.73 (s); HRMS-ESI m/z $[M+H]^+$ calcd for $C_{20}H_{22}N_9O$: 404.1947, found: 404.1913.

***N*-(4-(2,5-Diazabicyclo[2.2.1]heptan-2-yl)phenyl)-4-(thieno[2,3-d]pyrimidin-4-ylamino)-1*H*-pyrazole-3-carboxamide** (**8i**). Compound **8i** was prepared according to procedure F on 0.5 mmol scale. Purification by column chromatography (5% MeOH/DCM) yield the title compound (108 mg,

0.25 mmol, Yield 50%). White solid; m.p 249–250 °C. HPLC analysis: retention time = 3.345 min; peak area, 98.41%. ^{1}H NMR (300 MHz, DMSO-d_6) δ 13.55 (s, 1H, -NH-, pyrazole), 10.13 (s, 1H, -NHCO-), 10.08 (s, 1H, -NH-), 8.61 (s, 1H, ArH, pyrazole), 8.54 (s, 1H, ArH, Pyrimidine), 7.79 (d, *J* = 6.0 Hz, 1H, ArH, Thiophene), 7.68 (d, *J* = 8.9 Hz, 2H, ArH), 7.48 (d, *J* = 6.0 Hz, 1H, ArH, Thiophene), 6.67 (d, *J* = 9.0 Hz, 2H, ArH), 4.61 (s, 1H, -CH-), 4.44 (s, 1H, -CH-), 3.65-3.60 (m, 1H, -NH-), 3.28-3.16 (m, 2H, -CH_2-), 3.03-3.10 (m, 1H, -CH-), 2.15 (d, *J* = 10.9 Hz, 1H, -CH_2-), 1.93 (d, *J* = 11.8 Hz, -CH_2-); ^{13}C NMR (151 MHz, DMSO-d_6) δ 166.40 (s), 162.47 (s), 154.10 (s), 153.39 (s), 143.30 (s), 128.95 (s), 125.53 (s), 123.75 (s), 122.97 (s), 118.67 (s), 118.02 (s), 116.96 (s), 116.68 (s), 113.46 (s), 57.85 (s), 55.48 (s), 53.07 (s), 49.53 (s), 46.08 (s); HRMS-ESI m/z $[M+H]^+$ calcd for $C_{21}H_{21}N_8OS$: 433.1559, found: 433.1523.

***N*-(4-(2,5-Diazabicyclo[2.2.1]heptan-2-yl)phenyl)-4-((7*H*-pyrrolo[2,3-d]pyrimidin-4-yl)amino)-1*H*-pyrazole-3-carboxamide** (**8j**). Compound **8j** was prepared according to procedure F on 0.5 mmol scale. Purification by column chromatography (5% MeOH/DCM) yield the title compound (95 mg, 0.23 mmol, Yield 46 %). White solid; m.p 248–249 °C. HPLC analysis: retention time = 3.421 min; peak area, 97.56%. ^{1}H NMR (300 MHz, DMSO-d_6) δ 11.89 (s, 1H, -NH-, pyrrole), 10.01 (s, 1H, -NHCO-), 9.62 (s, 1H, -NH-), 8.55 (s, 1H, ArH, pyrazole), 8.38 (s, 1H, ArH, Pyrimidine), 7.62 (d, *J* = 8.9 Hz, 2H, ArH), 7.31 (d, *J* = 3.5 Hz, 1H, ArH, pyrrole), 6.60 (d, *J* = 8.9 Hz, 2H, ArH), 6.47 (d, *J* = 3.0 Hz, 1H, ArH, pyrrole), 4.43 (s, 1H, -CH-), 3.95 (s, 1H, -CH-), 3.55 (d, *J* = 8.1 Hz, 1H, -NH-), 3.02 – 2.94 (m, 4H, -CH_2-×2), 1.93 (d, *J* = 9.4 Hz, 1H, -CH_2-), 1.78 (d, *J* = 9.8 Hz, 1H, -CH_2-); ^{13}C NMR (151 MHz, DMSO-d_6) δ 162.52 (s), 152.12 (s), 151.68 (s), 150.98 (s), 144.04 (s), 132.38 (s), 128.20 (s), 124.92 (s), 123.46 (s), 122.95 (s), 121.04 (s), 113.13 (s), 103.51 (s), 97.17 (s), 56.82 (s), 56.27 (s), 49.04 (s), 36.77 (s), 36.25 (s); HRMS-ESI m/z $[M+H]^+$ calcd for $C_{21}H_{22}N_9O$: 416.1947, found: 416.1940.

***N*-(5-(Piperazin-1-yl)pyridin-2-yl)-4-(thieno[2,3-d]pyrimidin-4-ylamino)-1*H*-pyrazole-3-carboxamide** (**8k**). Compound **8k** was prepared according to procedure F on 0.5 mmol scale. Purification by column chromatography (5% MeOH/DCM) yield the title compound (84 mg, 0.20 mmol, Yield 39%). White solid; m.p >300 °C. HPLC analysis: retention time = 3.394 min; peak area, 100.00%.^{1}H NMR (300 MHz, DMSO-d_6) δ 13.62 (s, 1H, -NH-, pyrazole), 9.75 (s, 1H, -NHCO-), 9.39 (s, 1H, -NH-), 8.54 (d, *J* = 26.6 Hz, 2H, ArH), 8.18–7.98 (m, 2H, ArH), 7.78 (d, *J* = 6.0 Hz, 1H, ArH), 7.53 (d, *J* = 4.6 Hz, 2H, ArH), 3.43 (s, 4H, -CH2-×2), 3.06 (d, *J* = 5.1 Hz, 5H, -CH2-×2，-CH-); ^{13}C NMR (151 MHz, DMSO-d_6) δ 166.54 (s), 154.03 (s), 144.08 (s), 143.69 (s), 136.71 (s), 133.62 (s), 126.23 (s), 125.48 (s), 123.35 (s), 122.44 (s), 118.31 (s), 116.99 (s), 114.89 (s), 111.57 – 111.08 (m), 45.81 (d, *J* = 9.0 Hz), 8.89 (s); HRMS-ESI m/z $[M+H]^+$ calcd for $C_{19}H_{20}N_9OS$: 422.1512, found: 422.1521.

4-((7*H*-Pyrrolo[2,3-d]pyrimidin-4-yl)amino)-*N*-(5-(piperazin-1-yl)pyridin-2-yl)-1*H*-pyrazole-3-carboxamide (**8l**). Compound **8l** was prepared according to procedure F on 0.5 mmol scale. Purification by column chromatography (5% MeOH/DCM) yield the title compound (77 mg, 0.19 mmol, Yield 37%). white solid; m.p 288–290 °C. HPLC analysis: retention time = 3.085 min; peak area, 100.00%. ^{1}H NMR (300 MHz, DMSO-d_6) ^{1}H NMR (300 MHz, DMSO-d_6) δ13.51 (s, 1H, -NH-, pyrazole), 11.49 (s, 1H, -NH-, pyrrole)，9.57 (s, 1H, -NHCO-), 9.36 (s, 1H, -NH-), 9.29 (s, 1H, ArH), 8.51 (s, 1H, ArH), 8.36 (s, 1H, ArH), 8.05 (d, *J* = 8.1 Hz, 2H, ArH), 7.46 (d, *J* = 8.8 Hz, 1H, ArH), 7.28 (d, *J* = 3.0 Hz, 1H, ArH), 6.50 (d, *J* = 3.1 Hz, 1H, ArH), 3.53 (s, 4H, -CH_2-×2), 3.07 (s, 4H, -CH_2-×2); ^{13}C NMR (151 MHz, DMSO-d_6) δ 162.02 (s), 152.29 (s), 151.66 (s), 151.06 (s), 145.26 (s), 135.84 (s), 125.29 (s), 125.16 (s), 123.51 (s), 114.80 (s), 103.55 (s), 100.00 (s), 97.32 (s), 49.74 (s), 45.88 (s); HRMS-ESI m/z $[M+H]^+$ calcd for $C_{19}H_{21}N_{10}O$: 405.1900, found: 405.1901.

***N*-(5-(Piperazin-1-yl)pyridin-2-yl)-4-((6,7,8,9-tetrahydro-5*H*-cyclohepta[4,5]thieno[2,3-d]pyrimidin-4-yl)amino)-1*H*-pyrazole-3-carboxamide** (**8m**). Compound 8m was prepared according to procedure F on 0.5 mmol scale. Purification by column chromatography (5% MeOH/DCM) yield the title compound (118 mg, 0.24 mmol, Yield 48%). White solid, m.p 270–271 °C. HPLC analysis: retention time = 4.178 min; peak area, 99.56%.^{1}H NMR (300 MHz, DMSO-d_6) δ 13.43 (s, 1H, -NH-, pyrazole), 9.92 (s, 1H, -NHCO-), 9.50 (s, 1H, -NH-), 8.60 (s, 1H, ArH, pyrazole), 8.51 (s, 1H, ArH, Pyrimidine), 8.09 (m, 2H, ArH), 7.57 (d, *J* = 7.5 Hz, 1H, ArH), 3.35 (s, 6H, -CH_2-×3), 3.22 (s, 4H, -CH_2-×2), 2.95 (s, 2H, -CH_2-), 2.05-1.65 (m, 6H, -CH_2-×3); ^{13}C NMR (151 MHz, DMSO-d_6) δ 164.29 (s), 162.28 (s), 152.65 (s), 152.46 (s), 143.85 (s), 137.98 (s), 136.60 (s), 131.75 (s), 131.38 (s), 126.12 (s), 124.52 (s),

121.82 (s), 117.25 (s), 114.71 (s), 46.56 (s), 43.46 (s), 30.99 (s), 29.22 (s), 28.80 (s), 27.26 (s), 26.50 (s); HRMS-ESI m/z $[M+H]^+$ calcd for $C_{24}H_{28}N_9OS$: 490.2138, found: 490.2092.

***N*-(5-(Piperazin-1-yl)pyridin-2-yl)-4-(quinazolin-4-ylamino)-1*H*-pyrazole-3-carboxamide** (**8n**). Compound **8n** was prepared according to procedure F on 0.5 mmol scale. Purification by column chromatography (5% MeOH/DCM) yield the title compound (75 mg, 0.18 mmol, Yield 36%). Gray solid; m.p > 300 °C. HPLC analysis: retention time = 3.473 min; peak area, 97.81%. ^{1}H NMR (300 MHz, DMSO-d_6) δ 8.74 (s, 1H), 8.54 (d, *J* = 9.7 Hz, 1H), 8.28 (s, 1H), 8.24–8.06 (m, 3H), 7.52 (s, 1H), 7.41 (s, 4H), 3.25 (s, 4H), 3.05 (s, 4H); HRMS-ESI m/z $[M+H]^+$ calcd for $C_{21}H_{22}N_9O$: 416.1947, found: 416.1943.

4-((6,7-Dihydro-5*H*-cyclopenta[4,5]thieno[2,3-d]pyrimidin-4-yl)amino)-*N*-(5-(piperazin-1-yl)pyridin-2-yl)-1*H*-pyrazole-3-carboxamide (**8o**). Compound 8o was prepared according to procedure F on 0.5 mmol scale. Purification by column chromatography (5% MeOH/DCM) yield the title compound (97 mg, 0.21 mmol, Yield 42%). White solid; m.p 268–269 °C. HPLC analysis: retention time = 4.657 min; peak area, 96.57%.^{1}H NMR (300 MHz, DMSO-d_6) δ 9.67 (s, 1H, -NHCO-), 9.49 (s, 1H, -NH-), 8.58 (s, 1H, ArH, pyrazole), 8.50 (s, 1H, ArH, Pyrimidine), 8.09 (s, 1H, ArH), 8.02 (d, *J* = 8.5 Hz, 1H, ArH), 7.53 (d, *J* = 7.3 Hz, 1H, ArH), 3.24 (s, 8H, -CH_2-×4), 3.03 (d, *J* = 17.3 Hz, 6H, -CH_2-×3); ^{13}C NMR (151 MHz, DMSO-d_6) δ 170.77 (s), 162.14 (s), 153.02 (s), 152.33 (s), 144.11 (s), 143.71 (s), 139.24 (s), 136.50 (s), 135.17 (s), 131.53 (s), 126.00 (s), 124.49 (s), 114.85 (s), 113.77 (s), 43.84 (s), 47.04 (s), 29.57 (d, *J* = 25.3 Hz), 28.79 (s), 27.97 (s); HRMS-ESI m/z $[M+H]^+$ calcd for $C_{22}H_{24}N_9OS$: 462.1825, found: 462.1771.

***N*-(5-(Piperazin-1-yl)pyridin-2-yl)-4-((5,6,7,8-tetrahydrobenzo[4,5]thieno[2,3-d]pyrimidin-4-yl)amino)-1*H*-pyrazole-3-carboxamide** (**8p**). Compound 8p was prepared according to procedure F on 0.5 mmol scale. Purification by column chromatography (5% MeOH/DCM) yield the title compound (100 mg, 0.21 mmol, Yield 42%). White solid; m.p 266–267 °C. HPLC analysis: retention time = 5.017 min; peak area, 96.15%. ^{1}H NMR (300 MHz, DMSO-d_6) δ 13.35 (s, 1H, -NH-, pyrazole), 9.74 (s, 1H, -NHCO-), 9.46 (s, 1H, -NH-), 8.61 (s, 1H, ArH, pyrazole), 8.50 (s, 1H, ArH, Pyrimidine), 8.11 (s, 1H, ArH), 8.03 (d, *J* = 8.8 Hz, 1H, ArH), 7.53 (d, *J* = 8.5 Hz, 1H, ArH), 3.31 (s, 2H, -CH_2-), 3.16 (s, 8H, -CH_2-×4), 2.83 (s, 2H, -CH_2-), 1.91 (s, 4H, -CH_2-×2); ^{13}C NMR (151 MHz, DMSO-d_6) δ 165.42 (s), 162.03 (s), 152.86 (s), 152.79 (s), 144.07 (s), 143.74 (s), 136.47 (s), 133.60 (s), 131.71 (s), 126.31 (s), 125.94 (s), 124.44 (s), 121.80 (s), 116.49 (s), 114.69 (s), 47.06 (s), 43.82 (s), 25.84 (s), 25.42 (s), 22.59 (s), 22.40 (s); HRMS-ESI m/z $[M+H]^+$ calcd for $C_{23}H_{26}N_9OS$: 476.1981, found: 476.1972.

4-((6,7-Dihydro-5*H*-cyclopenta[d]pyrimidin-4-yl)amino)-*N*-(5-(piperazin-1-yl)pyridin-2-yl)-1*H*-pyrazole-3-carboxamide (**8q**). Compound **8q** was prepared according to procedure F on 0.5 mmol scale. Purification by column chromatography (5% MeOH/DCM) yield the title compound (97 mg, 0.24 mmol, Yield 48%). White solid; m.p > 290 °C. HPLC analysis: retention time = 4.012 min; peak area, 99.56%. ^{1}H NMR (300 MHz, DMSO-d_6) δ 13.55 (s, 1H, -NH-, pyrazole), 9.54 (s, 1H, -NHCO-), 8.95 (s, 1H, -NH-), 8.56 (s, 1H, ArH, pyrazole), 8.48 (s, 1H, ArH, Pyrimidine), 8.14 (s, 1H, ArH), 8.05 (d, *J* = 8.8 Hz, 1H, ArH), 7.56 (d, *J* = 8.4 Hz, 1H, ArH), 3.39 (s, 4H, -CH_2-×2), 3.28 (s, 4H, -CH_2-×2), 2.87 (m, 4H, -CH_2-×2), 2.11 (m, 2H, -CH_2-); ^{13}C NMR (151 MHz, DMSO-d_6) δ 171.09 (s), 162.34 (s), 157.02 (s), 155.23 (s), 144.07 (s), 143.65 (s), 136.72 (s), 132.23 (s), 126.26 (s), 124.68 (s), 120.81 (s), 114.88 (s), 45.99 (s), 43.01 (s), 34.05 (s), 26.64 (s), 21.40 (s); HRMS-ESI m/z $[M+H]^+$ calcd for $C_{20}H_{24}N_9O$: 406.2104, found: 406.2067

***N*-(4-Morpholinophenyl)-4-((5,6,7,8-tetrahydrobenzo[4,5]thieno[2,3-d]pyrimidin-4-yl)amino)-1*H*-pyrazole-3-carboxamide** (**8r**). Compound **8r** was prepared according to procedure F on 0.5 mmol scale. Purification by column chromatography (5% MeOH/DCM) yield the title compound (124 mg, 0.26 mmol, Yield 51%). White solid; m.p 267–268 °C. HPLC analysis: retention time = 3.619 min; peak area, 98.26%. ^{1}H NMR (300 MHz, DMSO-d_6) δ 13.39 (s, 1H, -NH-, pyrazole), 10.05 (s, 1H, -NHCO-), 10.01 (s, 1H, -NH-), 8.62 (s, 1H, ArH, pyrazole), 8.51 (s, 1H, ArH, Pyrimidine), 7.67 (d, *J* = 9.0 Hz, 2H, ArH), 6.94 (d, *J* = 9.0 Hz, 2H, ArH), 3.76-3.73 (m, 4H, -CH_2-×2), 3.18 (s, 2H, -CH_2-), 3.13-3.01 (m, 4H, -CH_2-×2), 2.90-2.73 (m, 2H, -CH_2-), 1.90 (s, 4H, -CH_2-×2); ^{13}C NMR (151 MHz, DMSO-d_6) δ 171.82 (s), 162.15 (s), 159.24 (s), 158.35 (s), 148.18 (s), 132.69 (s), 130.91 (s), 124.47 (s), 121.98

(s), 121.26 (s), 116.08 (s), 104.38 (s), 52.46 (s), 49.20 (s), 46.76 (s), 45.27 (s), 32.64 (s), 25.90 (s); HRMS-ESI m/z $[M+H]^+$ calcd for $C_{24}H_{26}N_7O_2S$: 476.1869, found: 476.1826.

***N*-(4-(2,5-Diazabicyclo[2.2.1]heptan-2-yl)phenyl)-4-((6,7-dihydro-5H-cyclopenta[4,5]thieno[2,3-d]pyrimidin-4-yl)amino)-1*H*-pyrazole-3-carboxamide** (**8s**). Compound 8s was prepared according to procedure F on 0.5 mmol scale. Purification by column chromatography (5% MeOH/DCM) yield the title compound (109 mg, 0.23 mmol, Yield 45%). White solid; m.p > 280 °C. HPLC analysis: retention time = 4.167 min; peak area, 98.32%. ^{1}H NMR (300 MHz, DMSO-d_6) δ 10.03 (s, 1H, -NHCO-), 9.84 (s, 1H, -NH-), 8.55 (d, *J* = 28.9 Hz, 2H, ArH), 7.55 (s, 2H, ArH), 6.56 (s, 2H, ArH), 4.31 (s, 1H, -CH-), 3.64 (s, 1H, -CH-), 3.49 (s, 1H, -NH-), 3.17 (s, 2H, -CH_2-), 2.87 (s, 6H, -CH_2-×3), 1.90-1.67 (m, 4H, -CH_2-×2); ^{13}C NMR (151 MHz, DMSO-d_6) δ 165.38 (s), 162.46 (s), 153.01 (s), 152.88 (s), 144.63 (s), 133.50 (s), 132.85 (s), 127.61 (s), 126.49 (s), 124.15 (s), 123.11 (s), 121.28 (s), 116.52 (s), 112.95 (s), 58.69 (s), 56.85 (s), 56.50 (s), 49.41 (s), 37.38 (s), 25.89 (s), 25.45 (s), 22.61 (s); HRMS-ESI m/z $[M+H]^+$ calcd for $C_{24}H_{25}N_8OS$: 473.1872, found: 473.1853.

4-((6,7-Dihydro-5*H*-cyclopenta[4,5]thieno[2,3-d]pyrimidin-4-yl)amino)-*N*-(4-(piperazin-1-yl)phenyl)-1*H*-pyrazole-3-carboxamide (**8t**). Compound 8t was prepared according to procedure F on 0.5 mmol scale. Purification by column chromatography (5% MeOH/DCM) yield the title compound (92 mg, 0.20 mmol, Yield 40%). white solid; m.p 266–268 °C. HPLC analysis: retention time = 4.752 min; peak area, 98.86%. ^{1}H NMR (300 MHz, DMSO-d_6) δ 10.15 (s, 1H, -NHCO-), 10.03 (s, 1H, -NH-), 8.62 (s, 1H, ArH, pyrazole), 8.52 (s, 1H, ArH, Pyrimidine), 7.67 (d, *J* = 7.1 Hz, 2H, ArH), 6.94 (d, *J* = 7.2 Hz, 2H, ArH), 3.13 (d, *J* = 18.0 Hz, 4H, -CH_2-×2), 2.95 (s, 4H, -CH_2-×2), 2.81 (s, 2H, -CH_2-), 1.89 – 1.63 (m, 4H, -CH_2-×2); ^{13}C NMR (151 MHz, DMSO-d_6) δ 172.58 (s), 165.40 (s), 162.80 (s), 152.90 (s), 147.53 (s), 139.18 (s), 133.58 (s), 131.18 (s), 129.55 (s), 126.46 (s), 124.31 (s), 122.56 (s), 122.48 (s), 116.56 (s), 49.06 (s), 47.40 (s), 45.95 (s), 43.88 (s), 21.62 (s); HRMS-ESI m/z $[M+H]^+$ calcd for $C_{23}H_{25}N_8OS$: 461.1872, found: 461.1823.

^{1}H NMR and ^{13}C NMR spectra

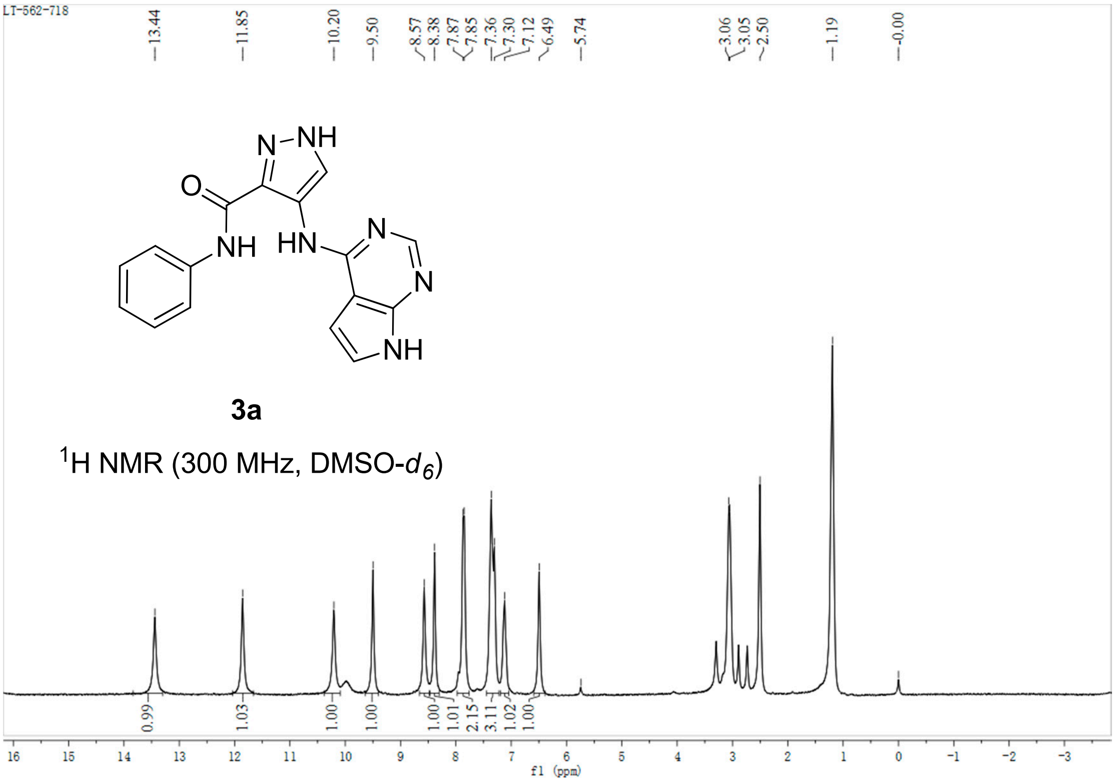

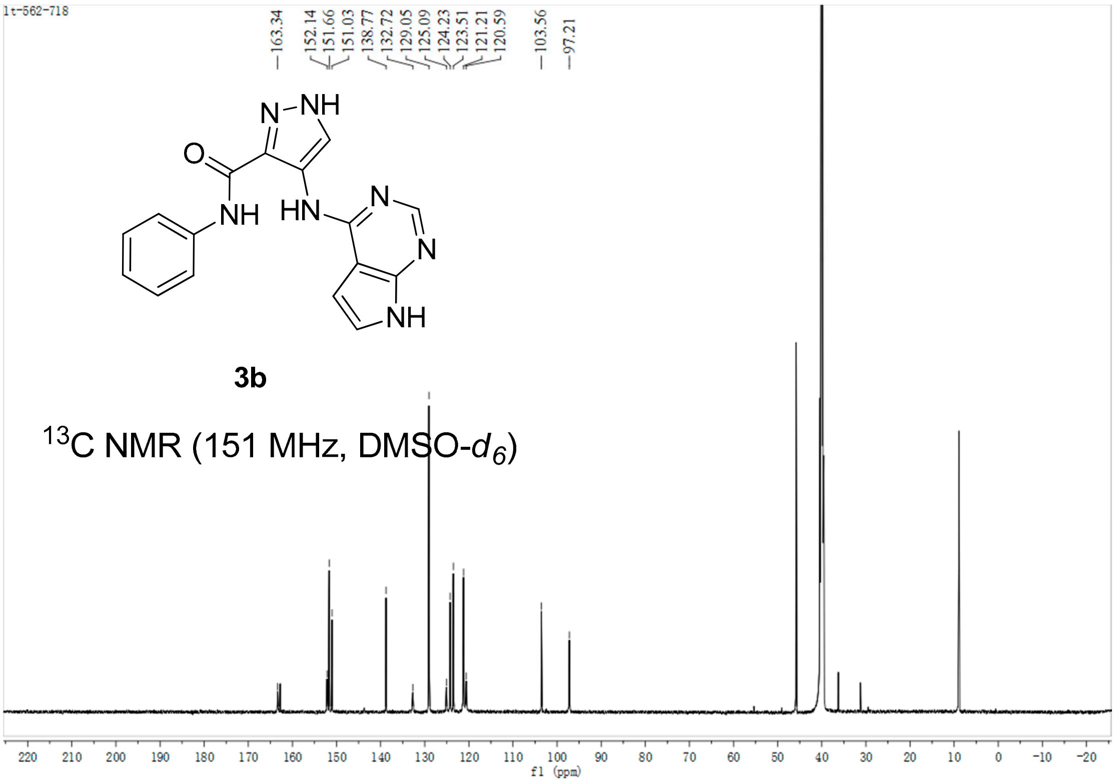

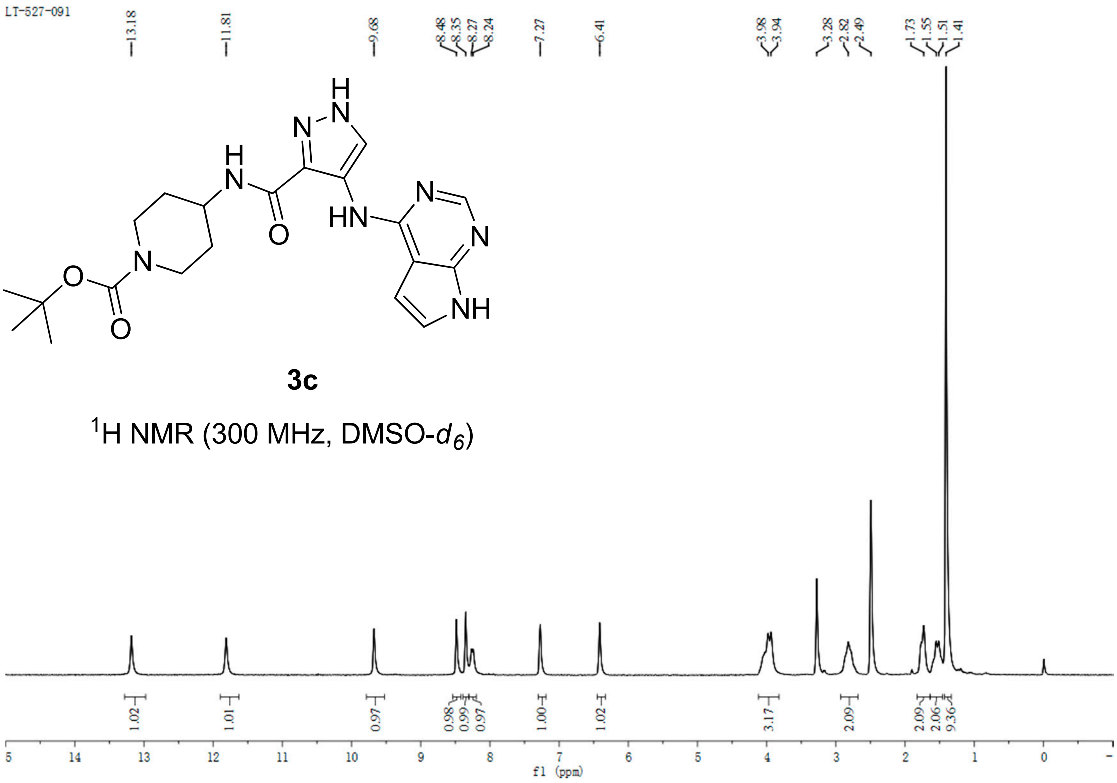
LT-527-091
13.18
11.81
9.68
8.48
8.35
8.27
8.24
7.27
6.41
3.98
3.94
3.28
2.82
2.49
1.73
1.55
1.51
1.41
3c
1H NMR (300 MHz, DMSO-d6)
1.02
1.01
0.97
0.98
0.99
0.97
1.00
1.02
3.17
2.09
2.09
2.06
9.36
f1 (ppm)

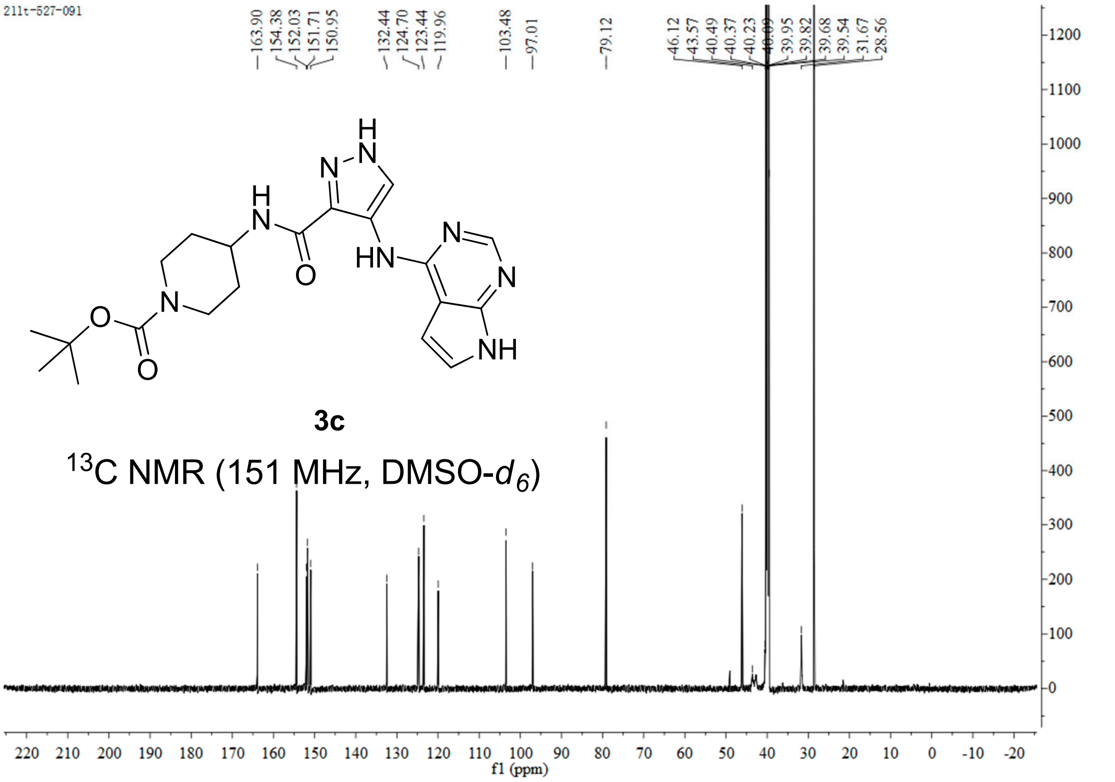
211t-527-091
163.90
154.38
152.03
151.71
150.95
132.44
124.70
123.44
119.96
103.48
97.01
79.12
46.12
43.57
40.49
40.37
40.23
39.95
39.82
39.68
39.54
31.67
28.56
3c
13C NMR (151 MHz, DMSO-d6)
f1 (ppm)

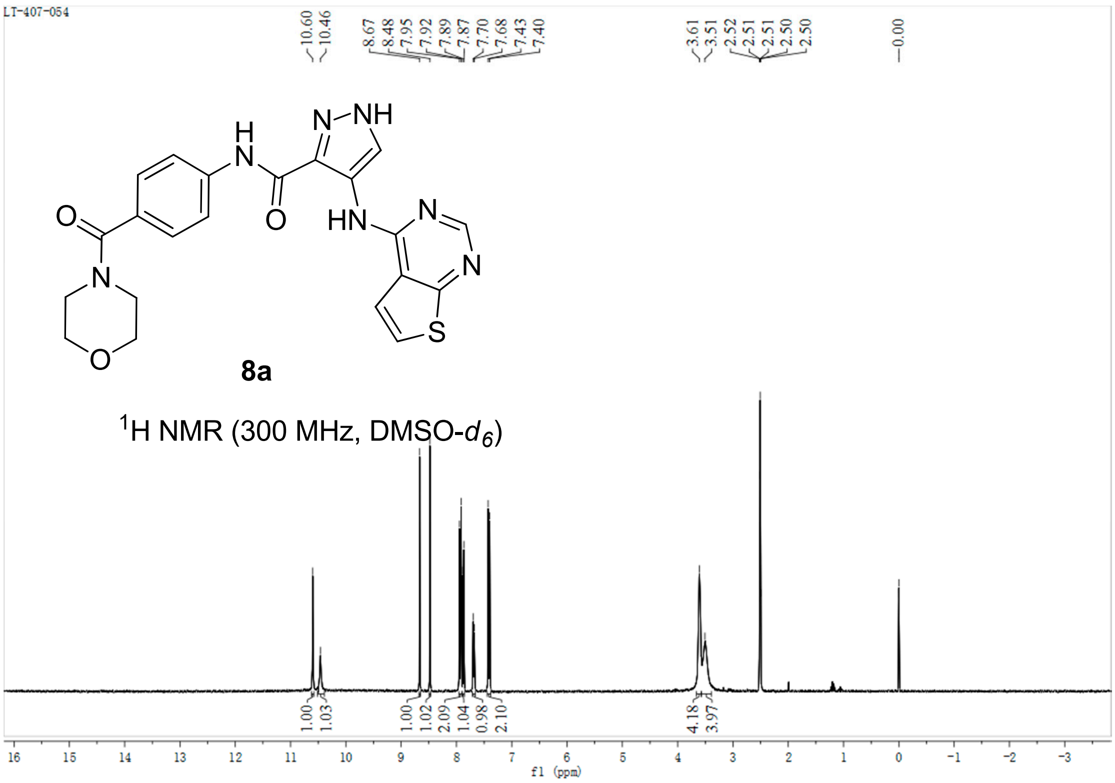
LT-407-054
10.60
10.46
8.67
8.48
7.95
7.92
7.89
7.87
7.70
7.68
7.43
7.40
3.61
3.51
2.52
2.51
2.51
2.50
2.50
0.00
8a
1H NMR (300 MHz, DMSO-d6)
1.00
1.03
1.00
1.02
2.09
1.04
0.98
2.10
4.18
3.97
f1 (ppm)

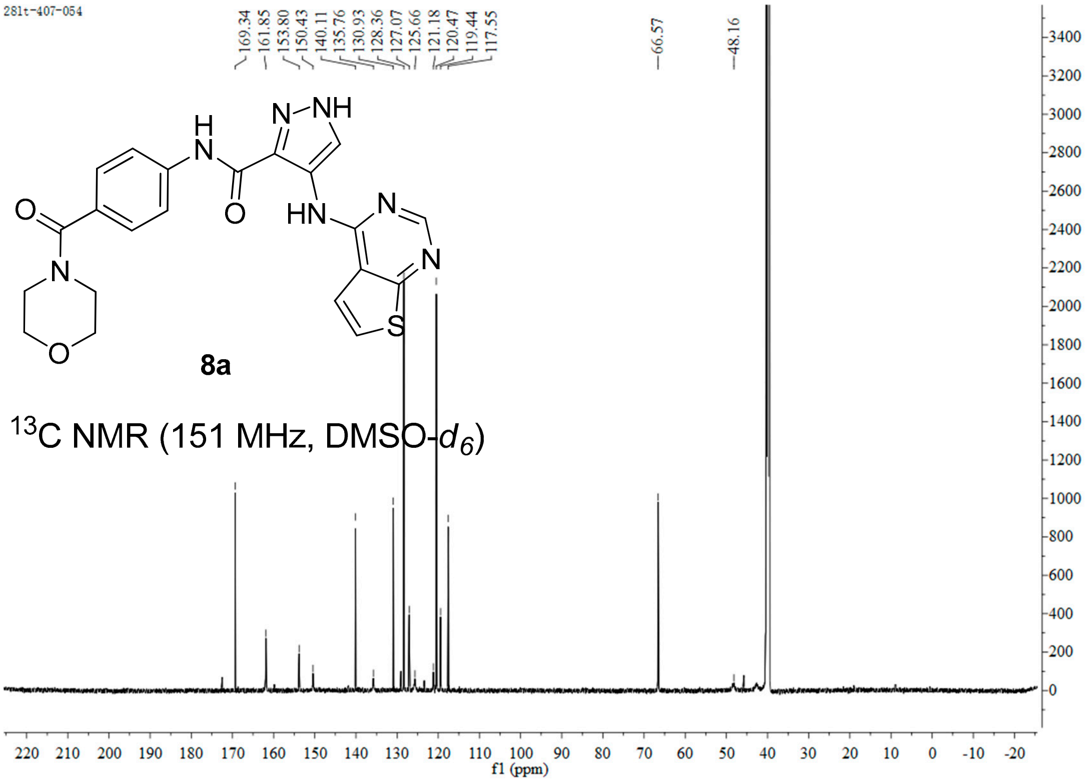
281t-407-054
169.34
161.85
153.80
150.43
140.11
135.76
130.93
128.36
127.07
125.66
121.18
120.47
119.44
117.55
66.57
48.16
8a
13C NMR (151 MHz, DMSO-d6)
f1 (ppm)

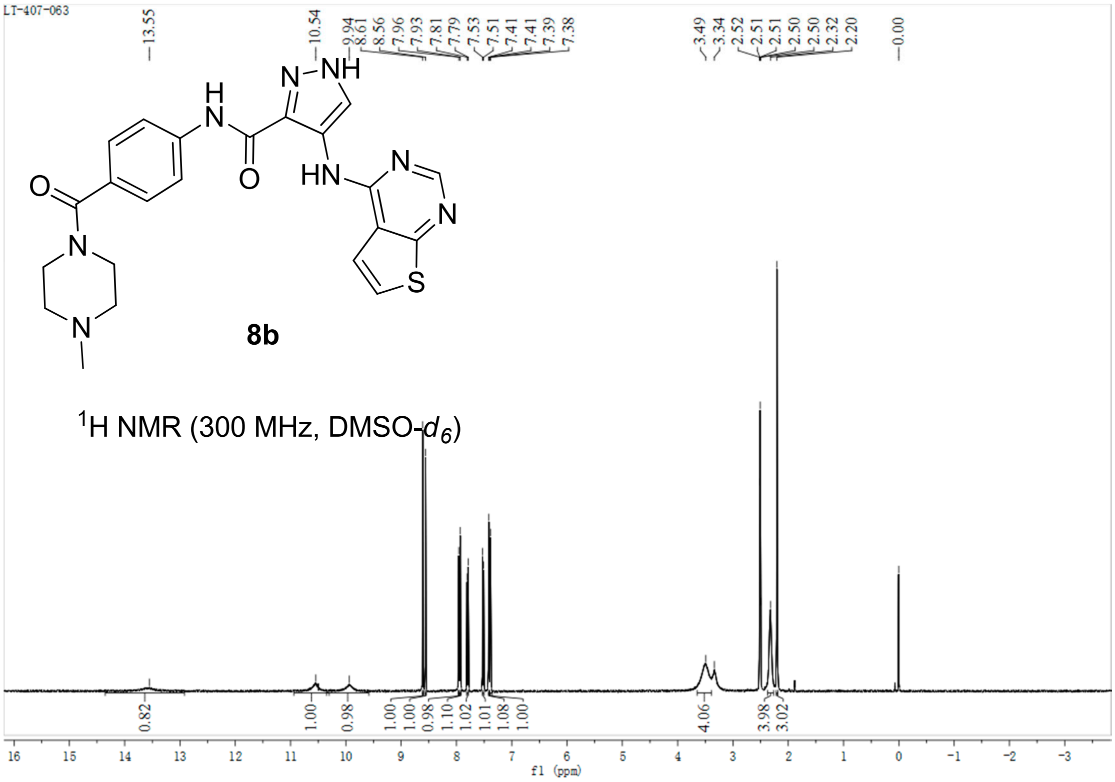
LT-407-063
13.55
10.54
9.94
8.61
8.56
7.96
7.93
7.81
7.79
7.53
7.51
7.41
7.41
7.39
7.38
3.49
3.34
2.52
2.51
2.51
2.50
2.50
2.32
2.20
0.00
8b
1H NMR (300 MHz, DMSO-d6)
0.82
1.00
0.98
1.00
1.00
0.98
1.10
1.02
1.01
1.08
1.00
4.06
3.98
3.02
f1 (ppm)

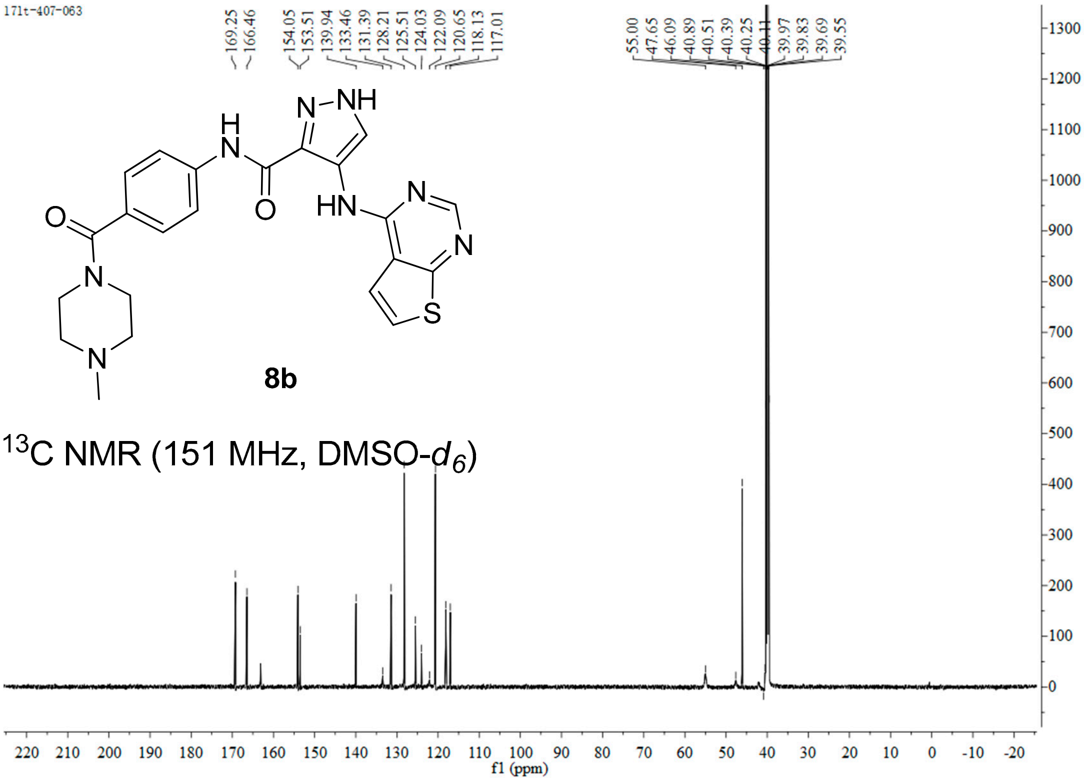
171t-407-063
169.25
166.46
154.05
153.51
139.94
133.46
131.39
128.21
125.51
124.03
122.09
120.65
118.13
117.01
55.00
47.65
46.09
40.89
40.51
40.39
40.25
40.11
39.97
39.83
39.69
39.55
8b
13C NMR (151 MHz, DMSO-d6)
f1 (ppm)

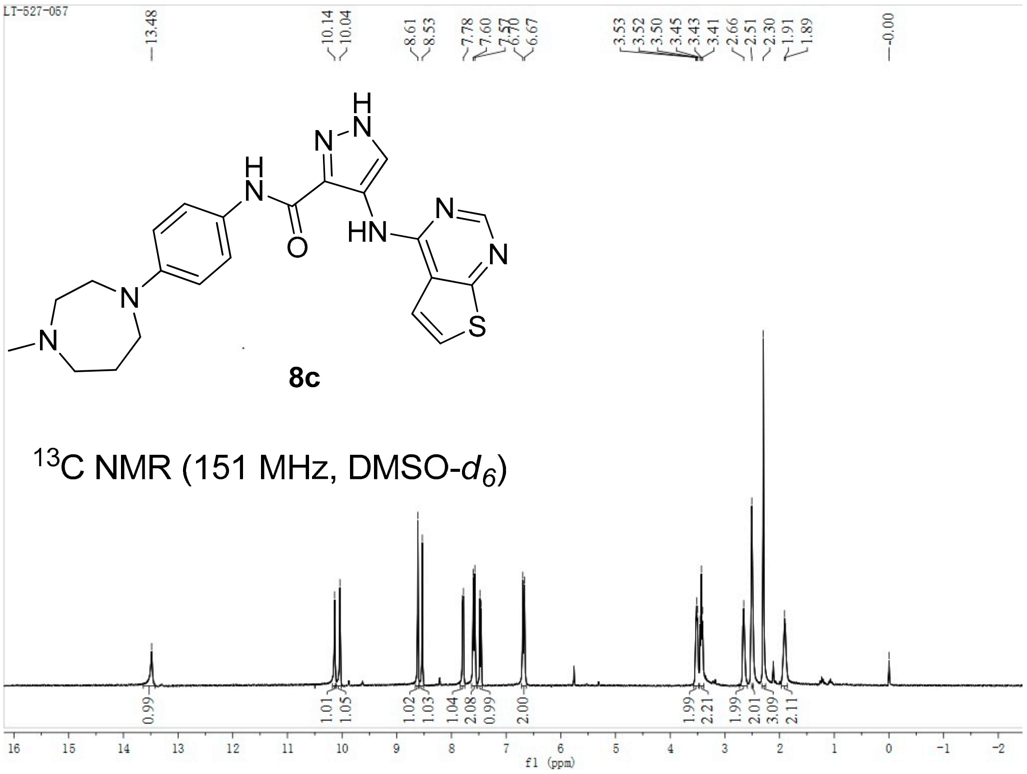
LT-527-057
8c
^{13}C NMR (151 MHz, DMSO-d_6)
f1 (ppm)

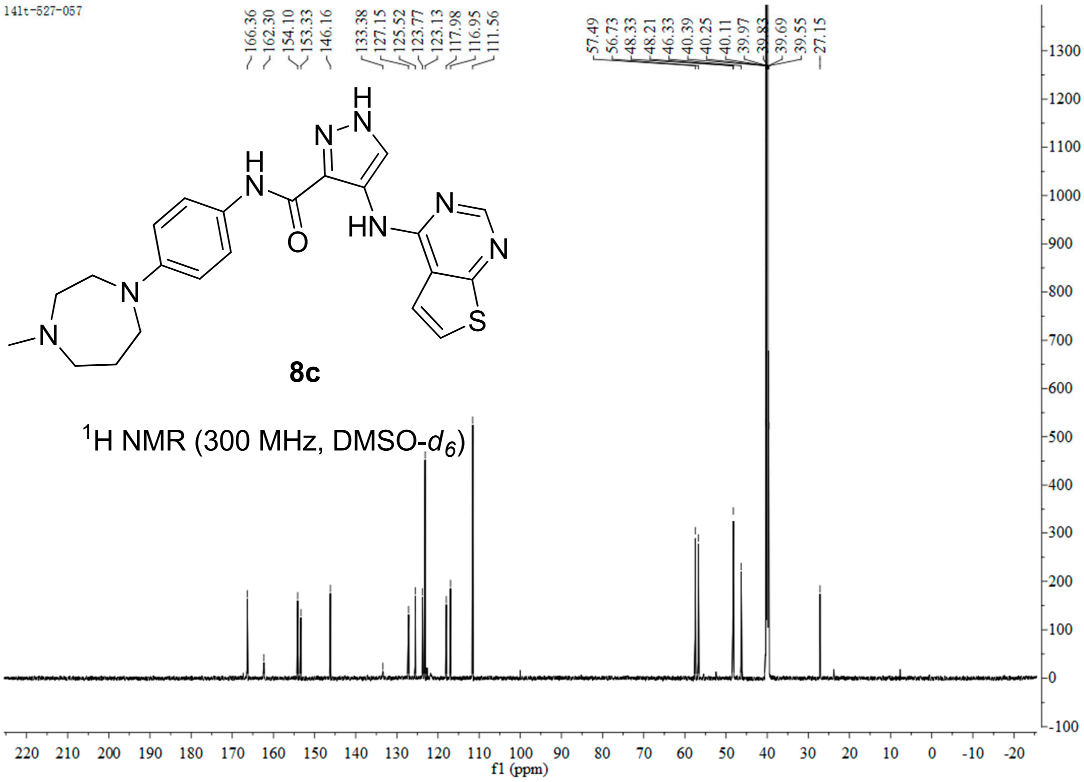
141t-527-057
8c
^{1}H NMR (300 MHz, DMSO-d_6)
f1 (ppm)

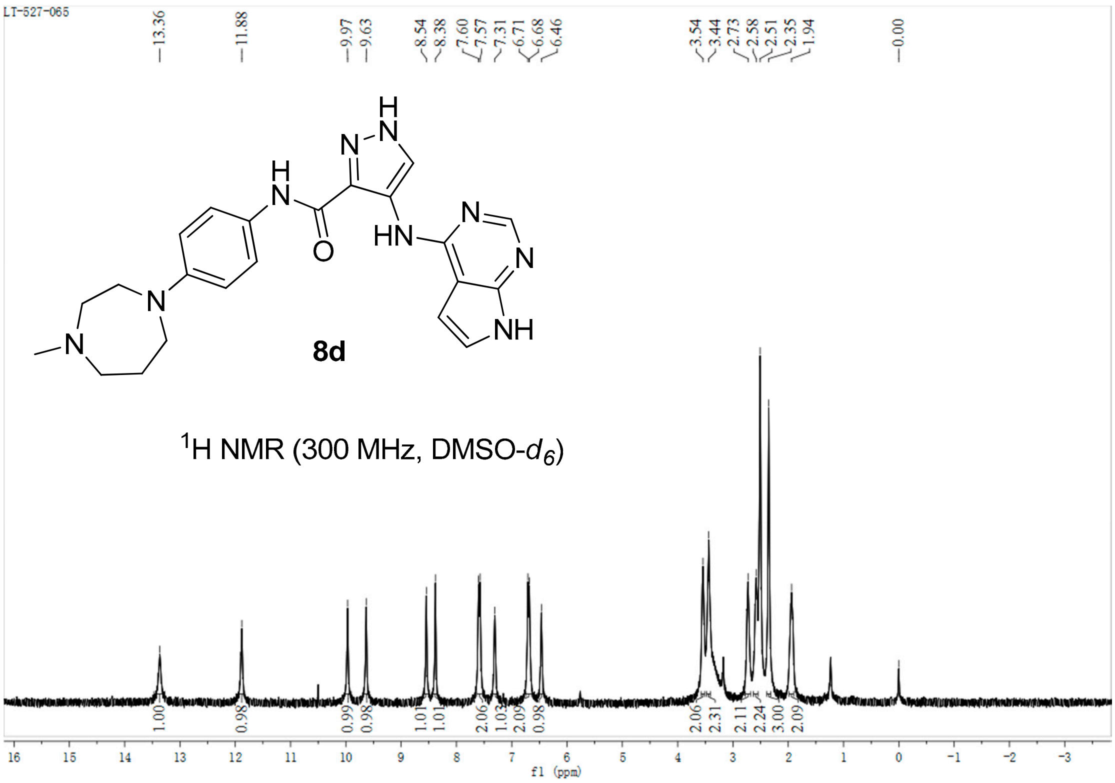
LT-527-065
8d
1H NMR (300 MHz, DMSO-d6)

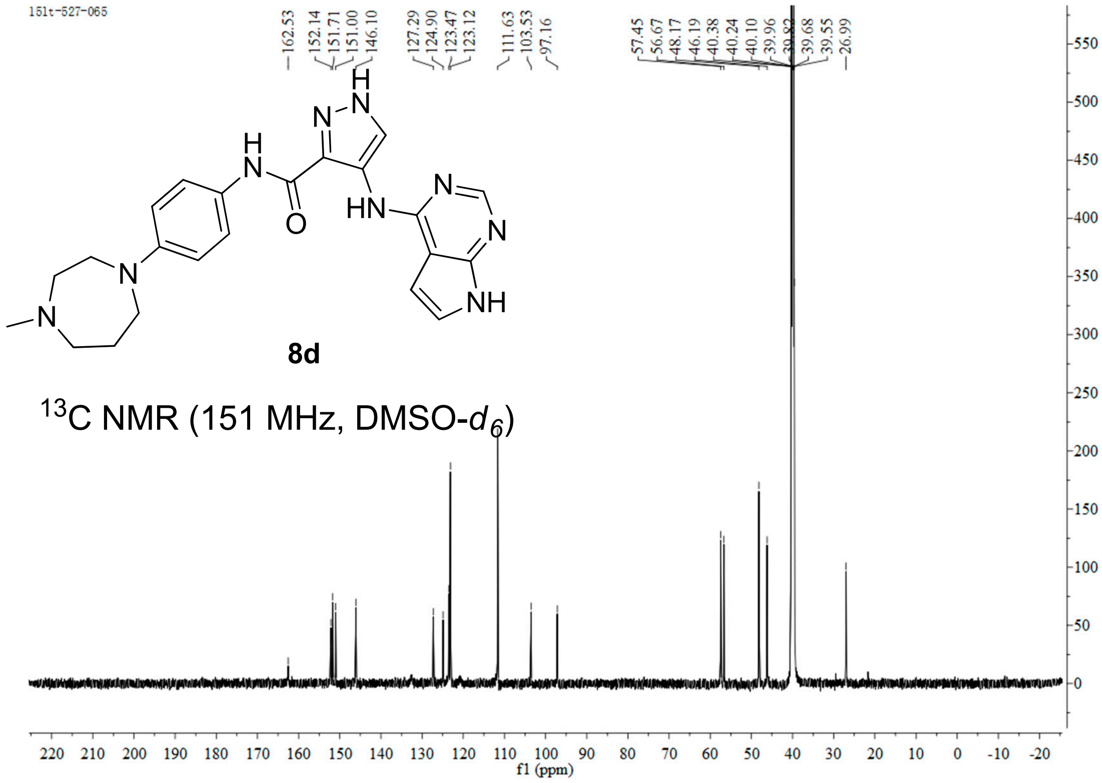
151t-527-065
8d
13C NMR (151 MHz, DMSO-d6)

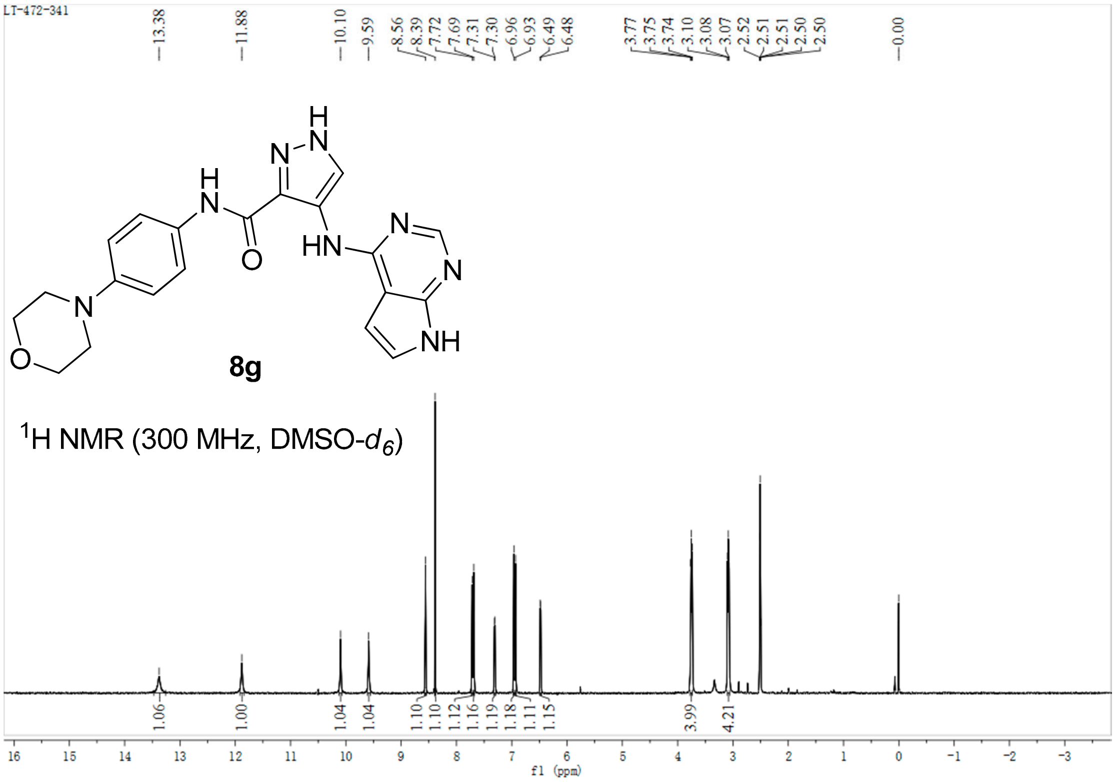
LT-472-341
13.38
11.88
10.10
9.59
8.56
8.39
7.72
7.69
7.31
7.30
6.96
6.93
6.49
6.48
3.77
3.75
3.74
3.10
3.08
3.07
2.52
2.51
2.51
2.50
2.50
0.00
8g
1H NMR (300 MHz, DMSO-d6)
1.06
1.00
1.04
1.04
1.10
1.10
1.12
1.16
1.19
1.18
1.11
1.15
3.99
4.21
f1 (ppm)

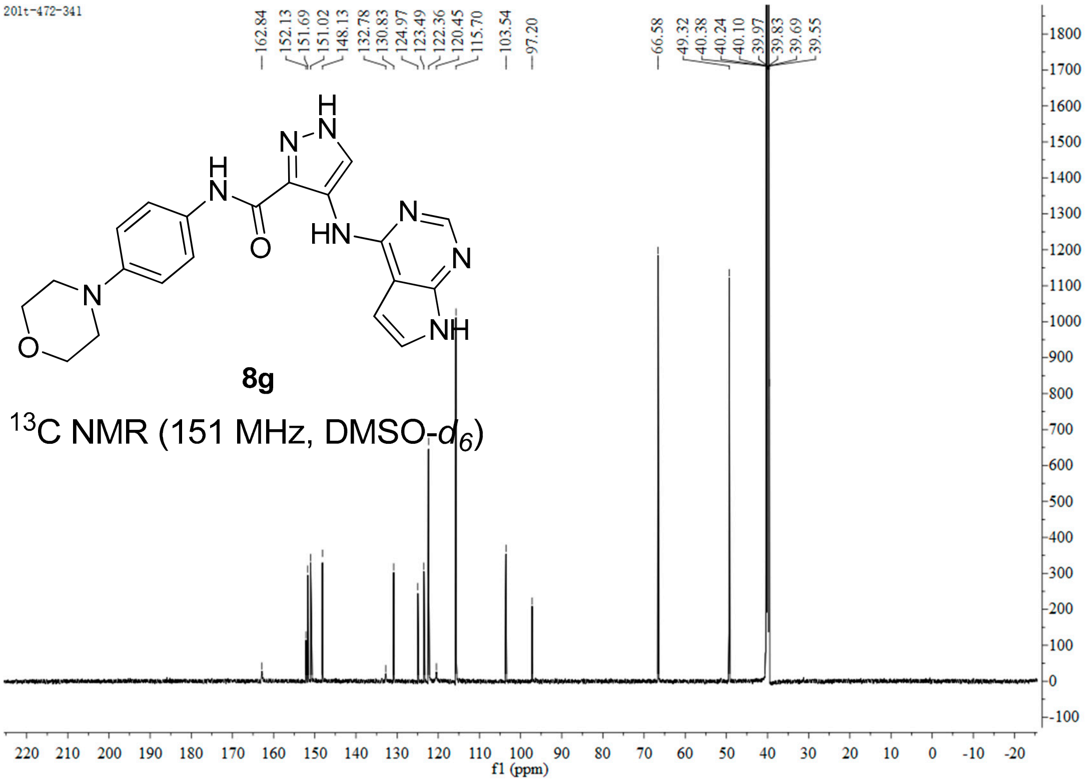
201t-472-341
162.84
152.13
151.69
151.02
148.13
132.78
130.83
124.97
123.49
122.36
120.45
115.70
103.54
97.20
66.58
49.32
40.38
40.24
40.10
39.97
39.83
39.69
39.55
8g
13C NMR (151 MHz, DMSO-d6)
f1 (ppm)

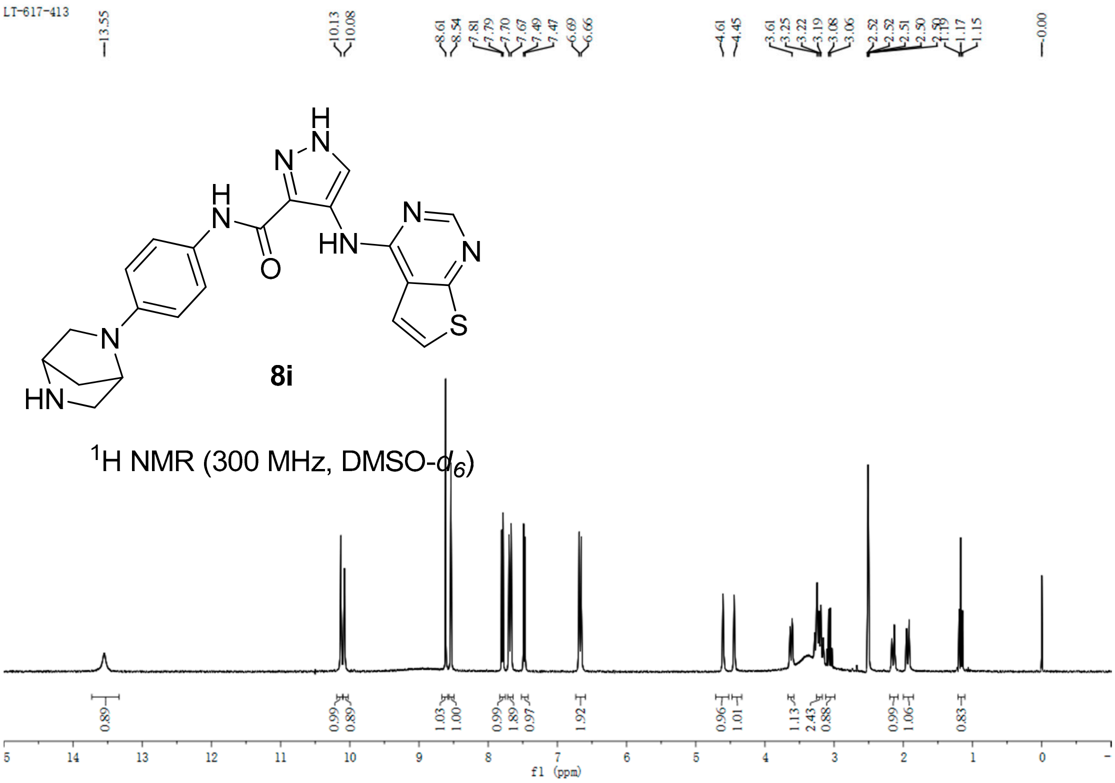
LT-617-413
8i
^{1}H NMR (300 MHz, DMSO-d_6)
f1 (ppm)

410

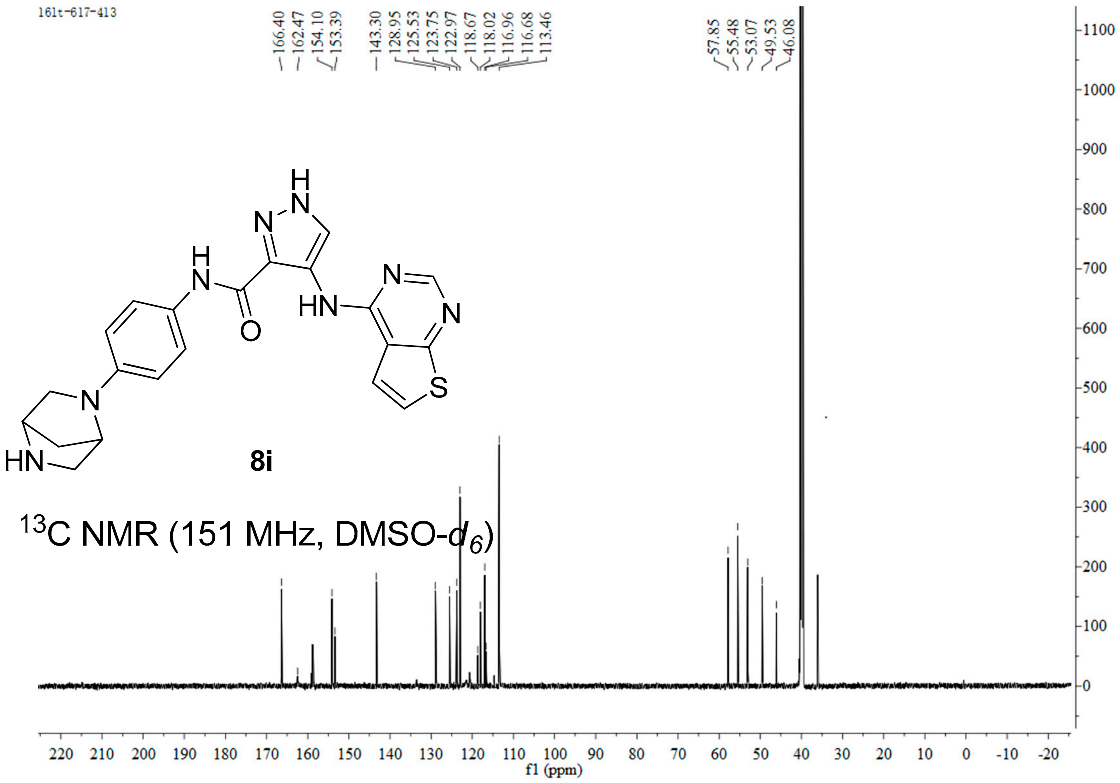
161t-617-413
8i
^{13}C NMR (151 MHz, DMSO-d_6)
f1 (ppm)

411

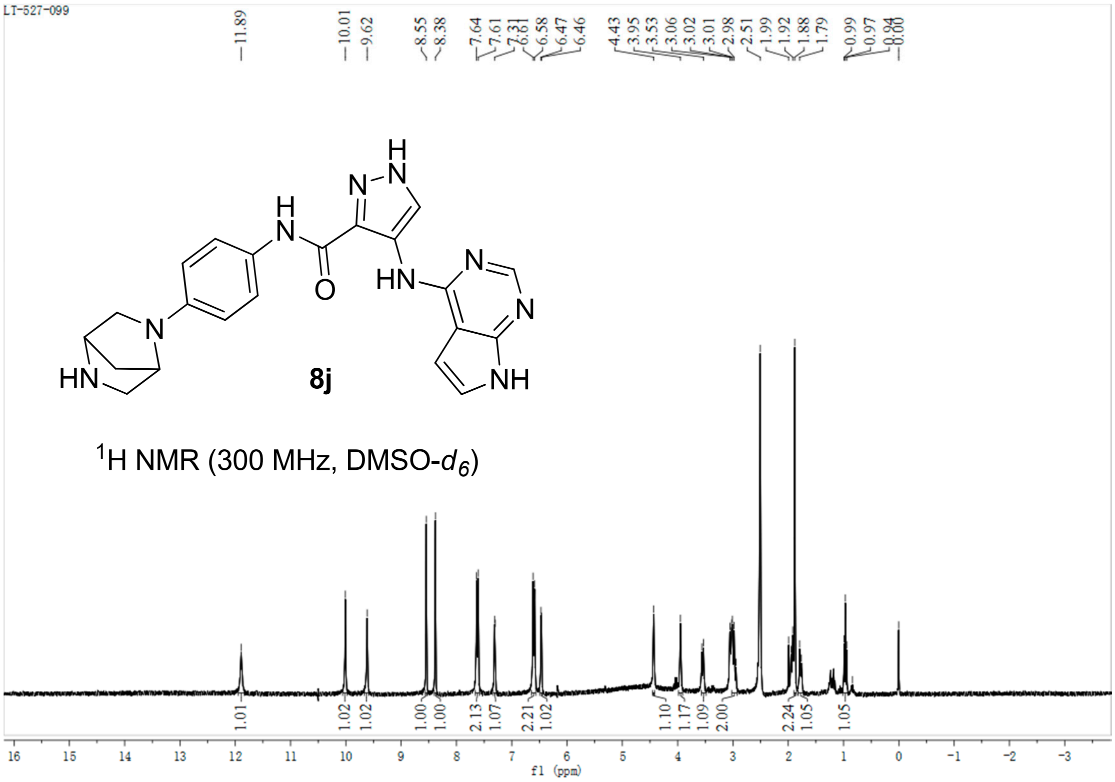
LT-527-099
8j
^{1}H NMR (300 MHz, DMSO-d_6)
f1 (ppm)

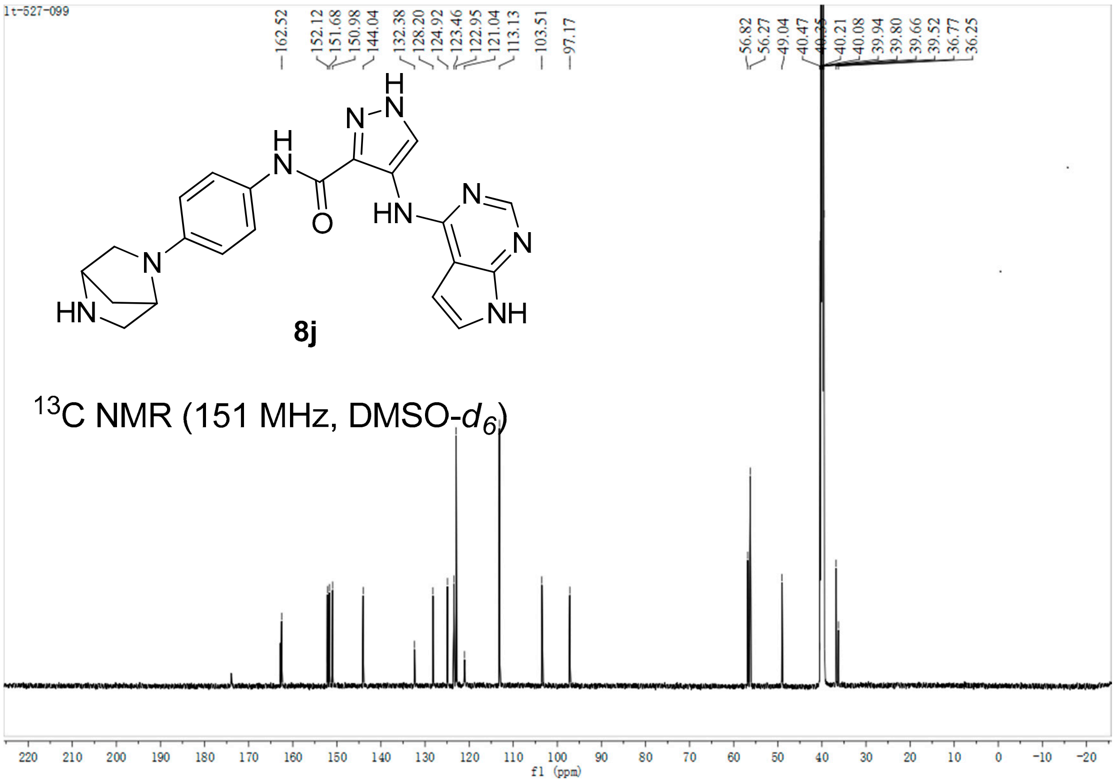
lt-527-099
8j
^{13}C NMR (151 MHz, DMSO-d_6)
f1 (ppm)

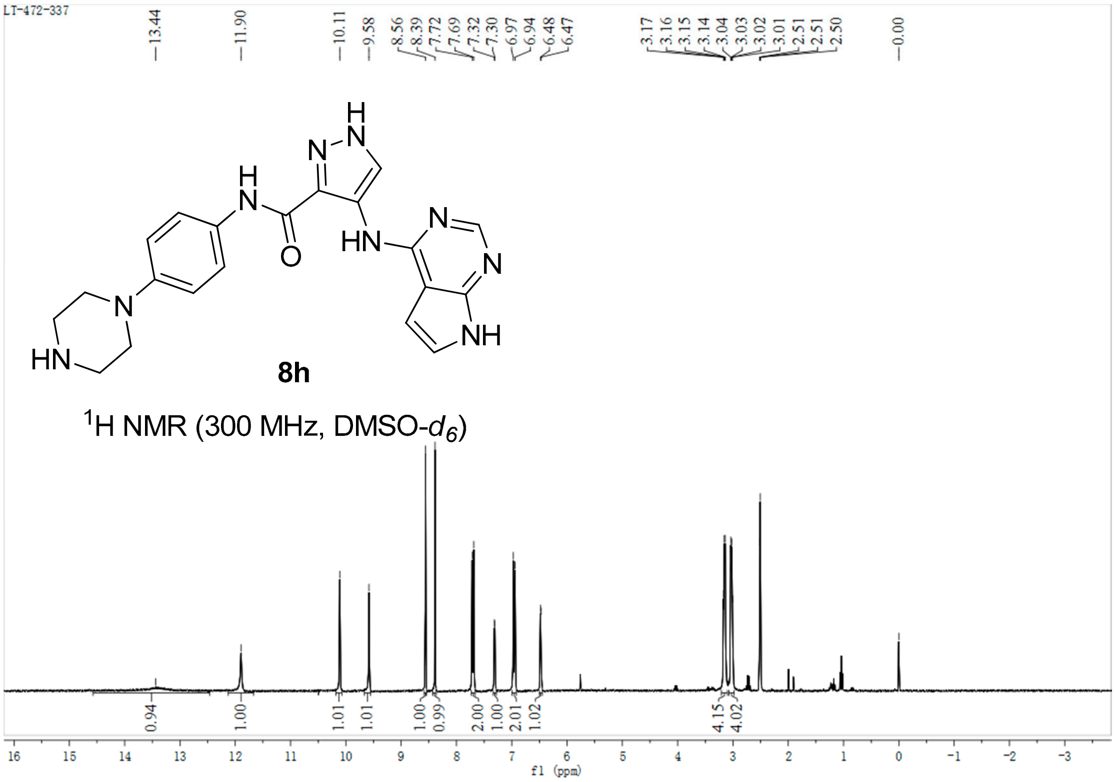
LT-472-337
8h
1H NMR (300 MHz, DMSO-d6)
f1 (ppm)

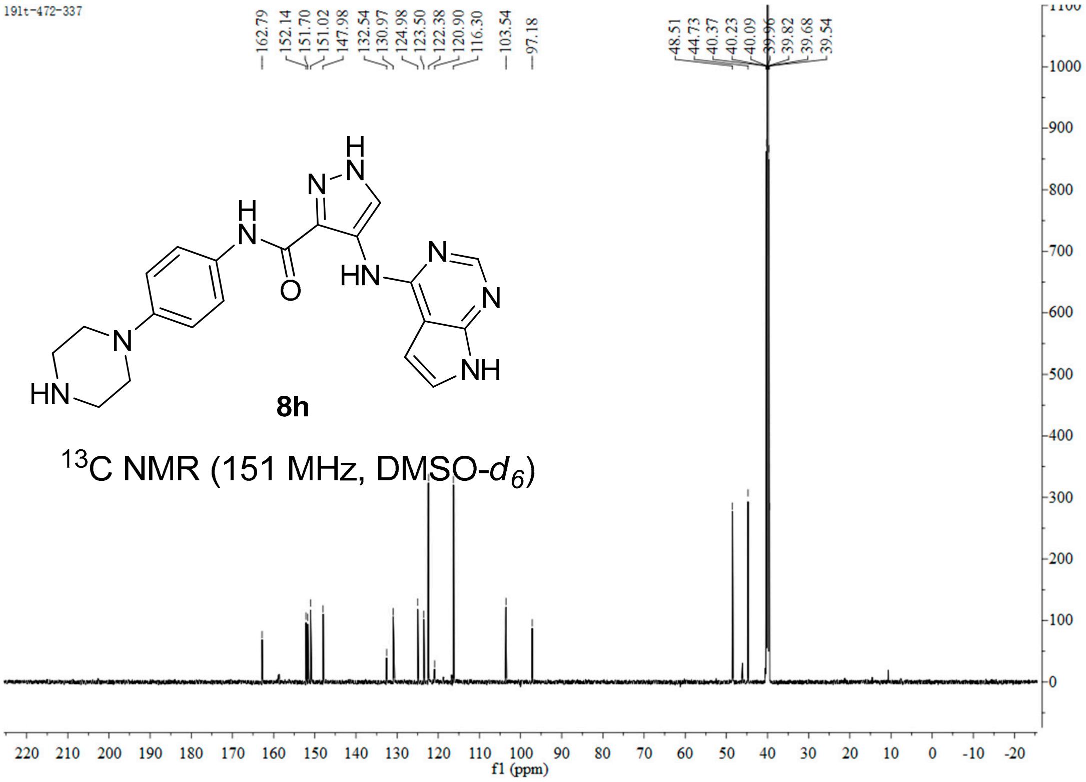
191t-472-337
8h
13C NMR (151 MHz, DMSO-d6)
f1 (ppm)

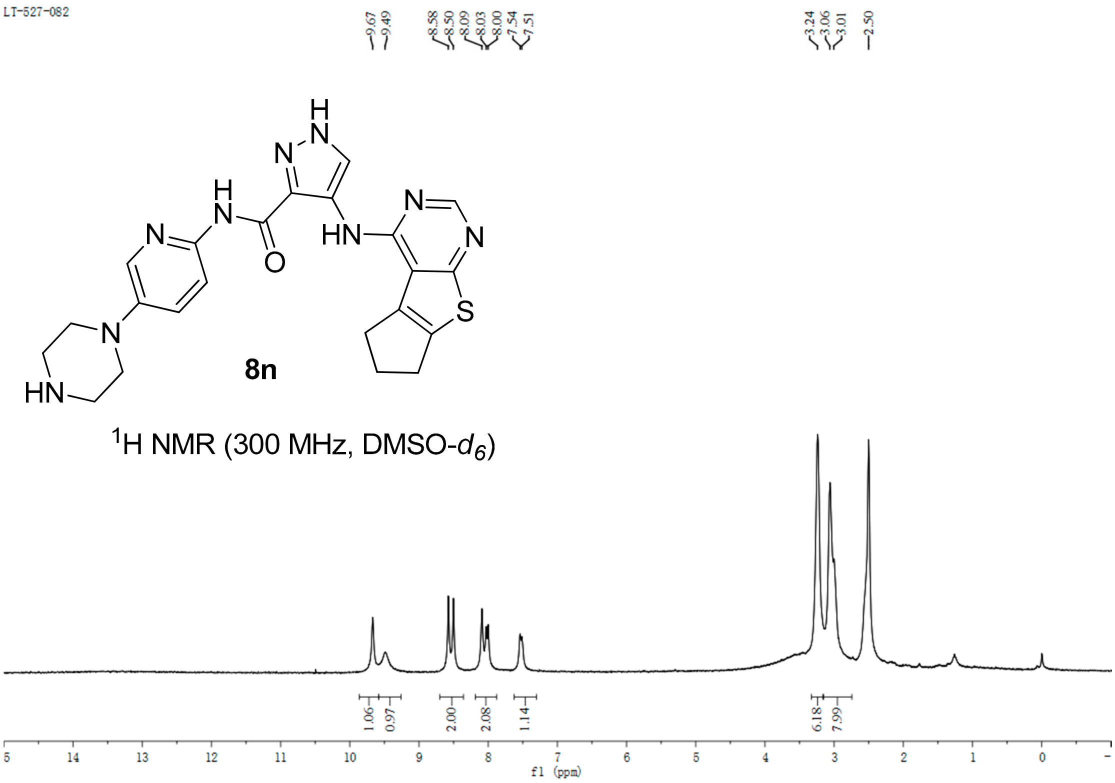
LT-527-082
8n
^{1}H NMR (300 MHz, DMSO-d_6)
f1 (ppm)

416

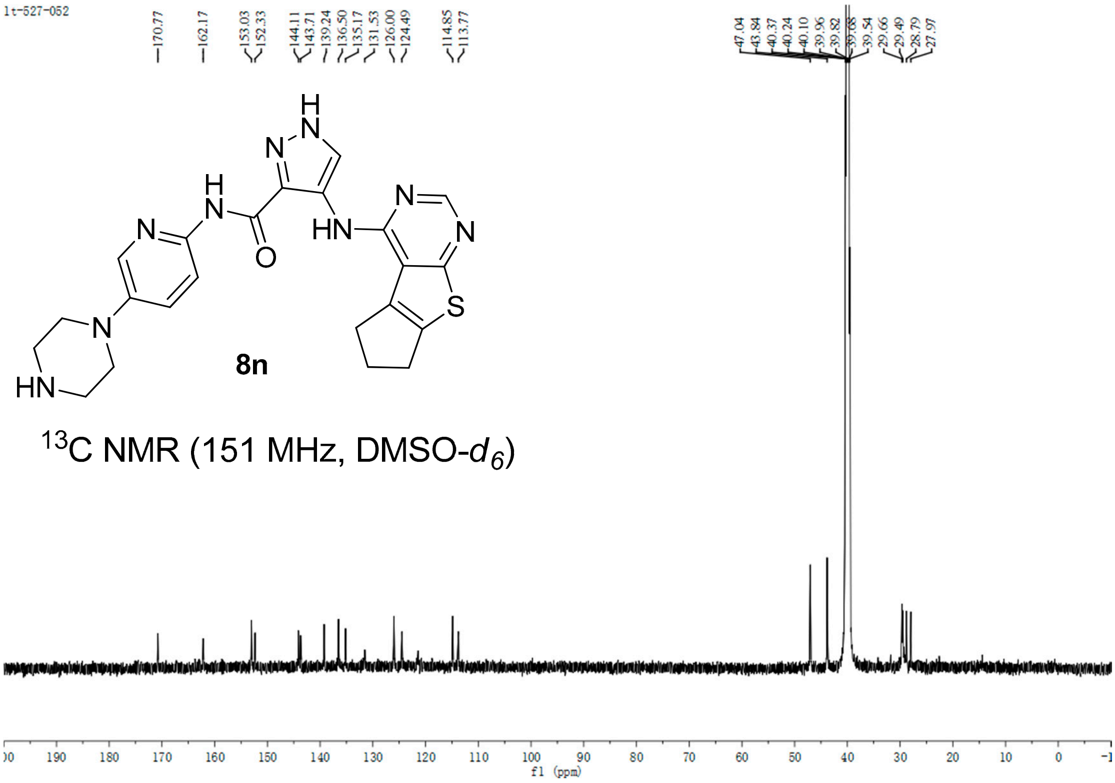
lt-527-052
8n
^{13}C NMR (151 MHz, DMSO-d_6)
f1 (ppm)

417

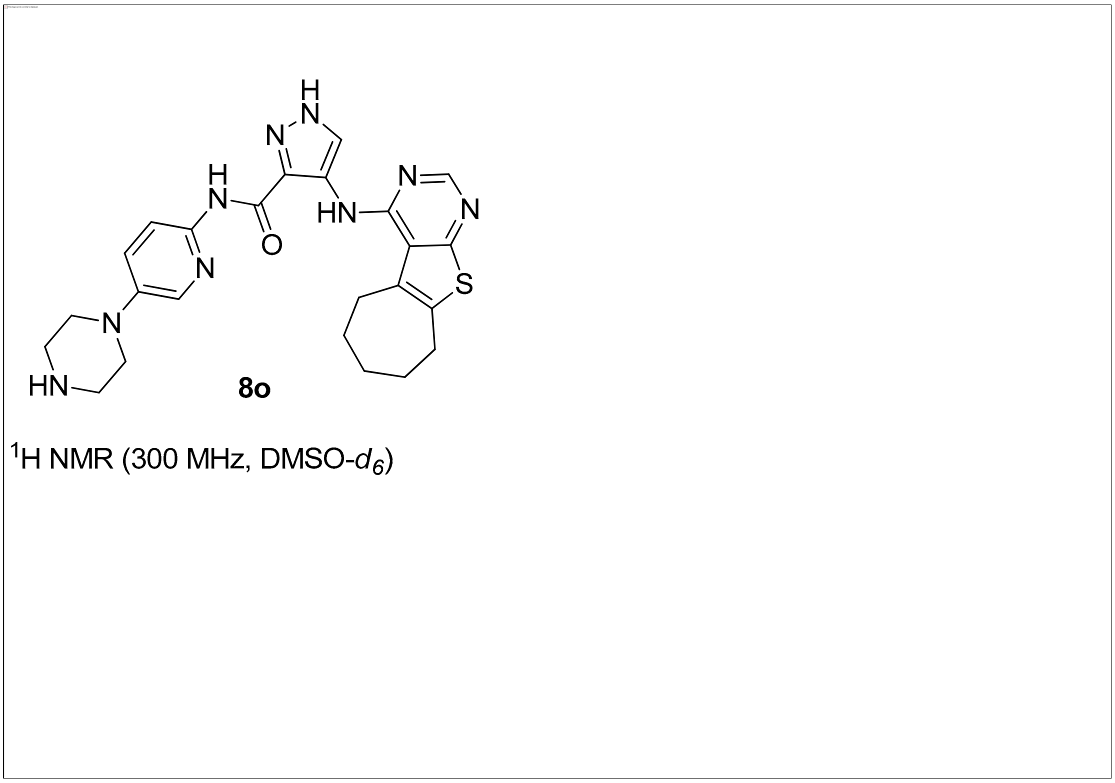
8o
1H NMR (300 MHz, DMSO-d6)

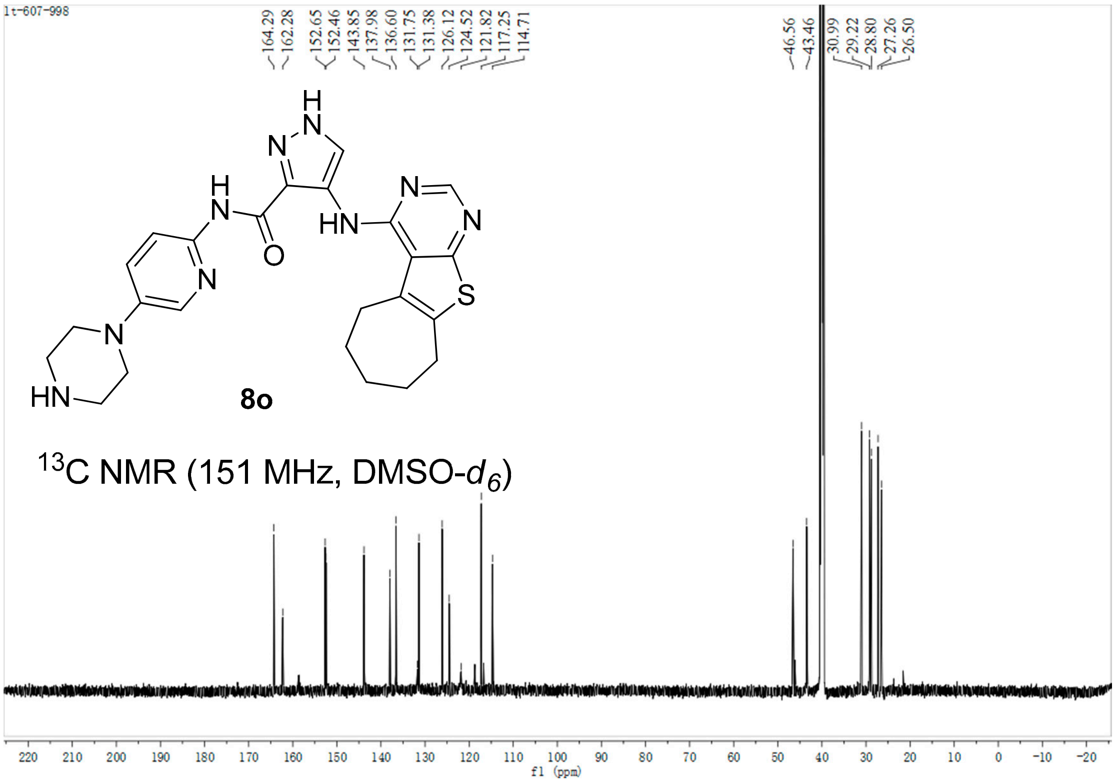
1t-607-998
164.29
162.28
152.65
152.46
143.85
137.98
136.60
131.75
131.38
126.12
124.52
121.82
117.25
114.71
46.56
43.46
30.99
29.22
28.80
27.26
26.50
8o
13C NMR (151 MHz, DMSO-d6)
220 210 200 190 180 170 160 150 140 130 120 110 100 90 80 70 60 50 40 30 20 10 0 -10 -20
f1 (ppm)

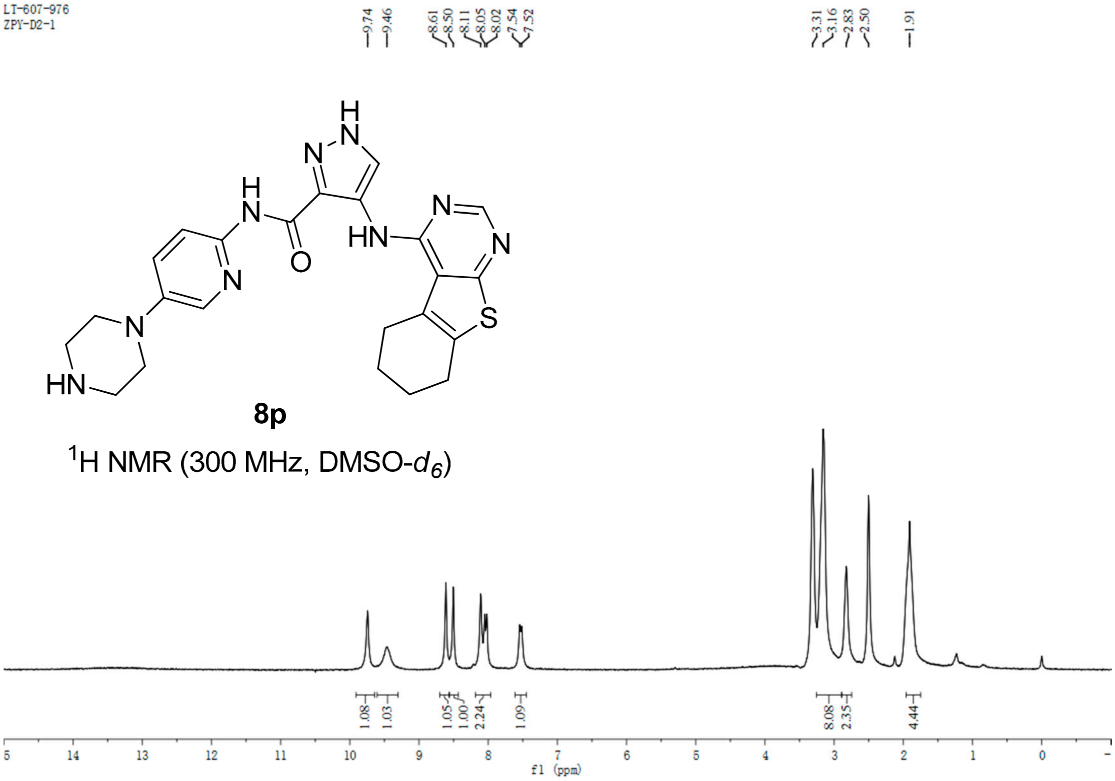
LT-607-976
ZPY-D2-1
8p
1H NMR (300 MHz, DMSO-d6)
f1 (ppm)

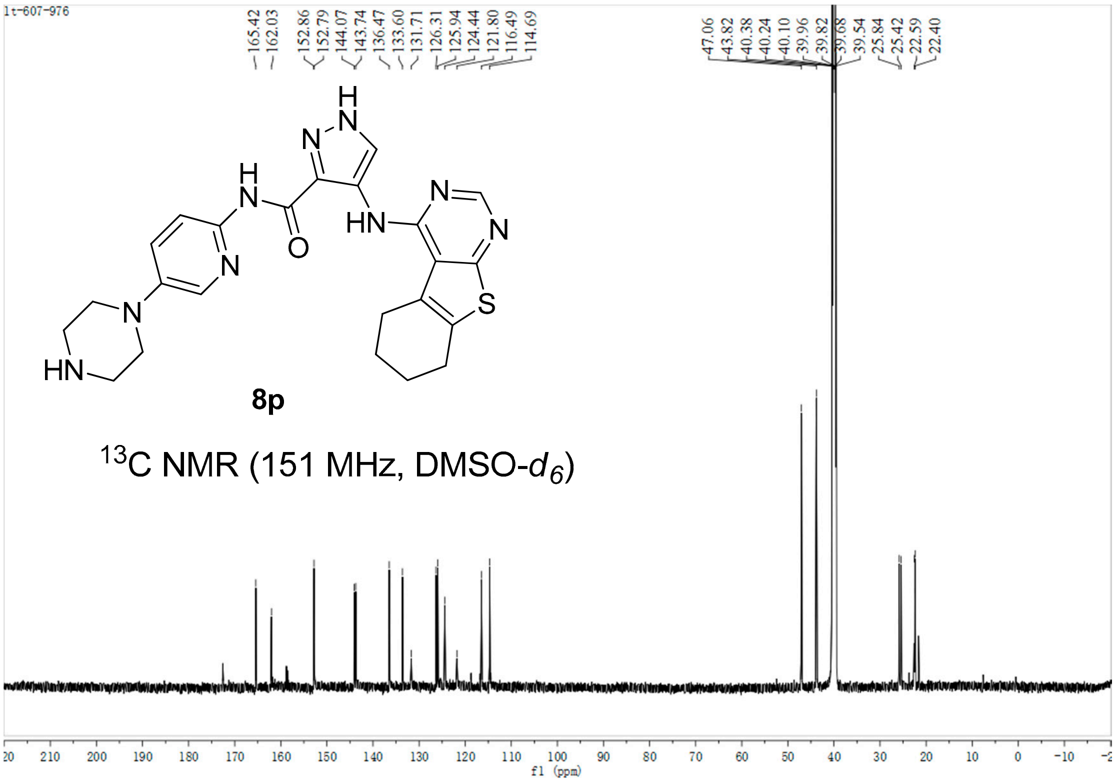
lt-607-976
8p
13C NMR (151 MHz, DMSO-d6)
f1 (ppm)

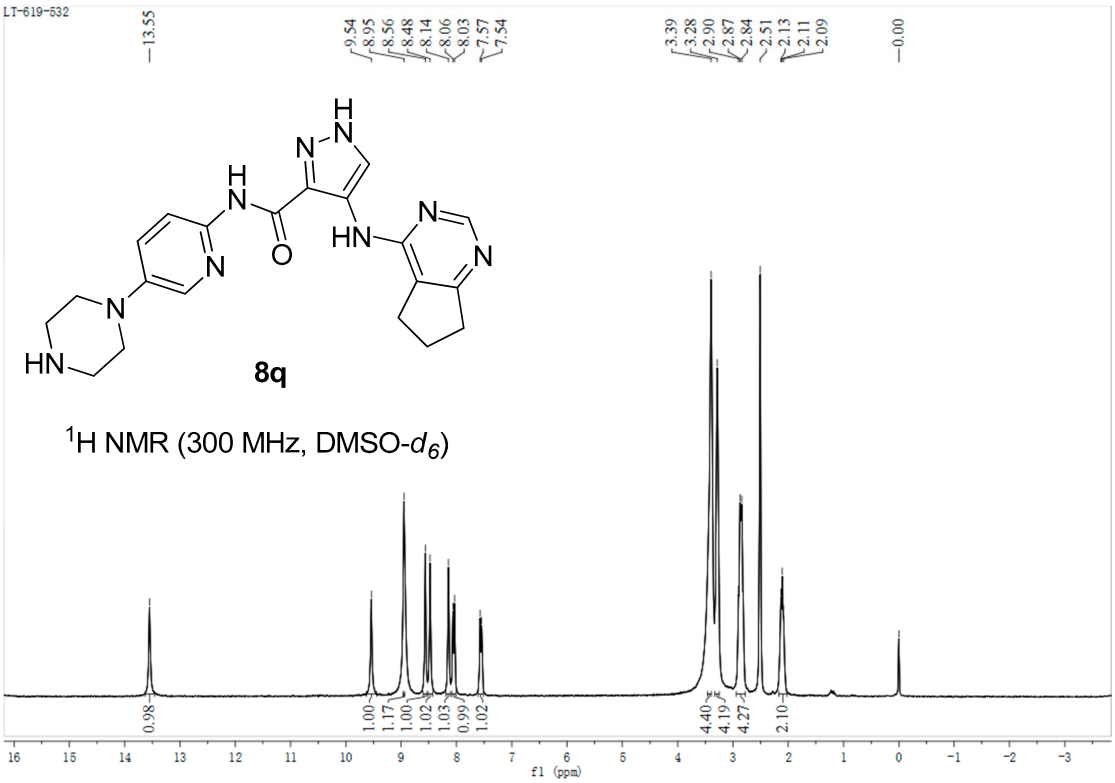
LT-619-532
13.55
9.54
8.95
8.56
8.48
8.14
8.06
8.03
7.57
7.54
3.39
3.28
2.90
2.87
2.84
2.51
2.13
2.11
2.09
0.00
8q
1H NMR (300 MHz, DMSO-d6)
0.98
1.00
1.17
1.00
1.02
1.03
0.99
1.02
4.40
4.19
4.27
2.10
f1 (ppm)

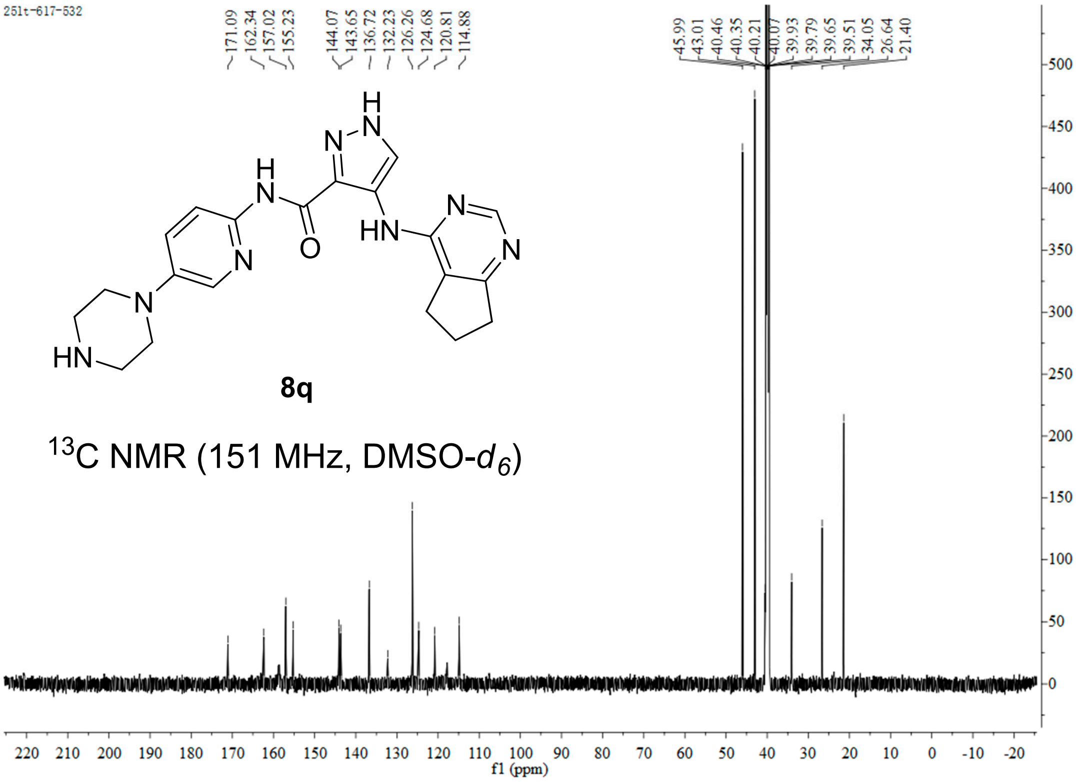
251t-617-532
171.09
162.34
157.02
155.23
144.07
143.65
136.72
132.23
126.26
124.68
120.81
114.88
45.99
43.01
40.46
40.35
40.21
40.07
39.93
39.79
39.65
39.51
34.05
26.64
21.40
8q
13C NMR (151 MHz, DMSO-d6)
f1 (ppm)

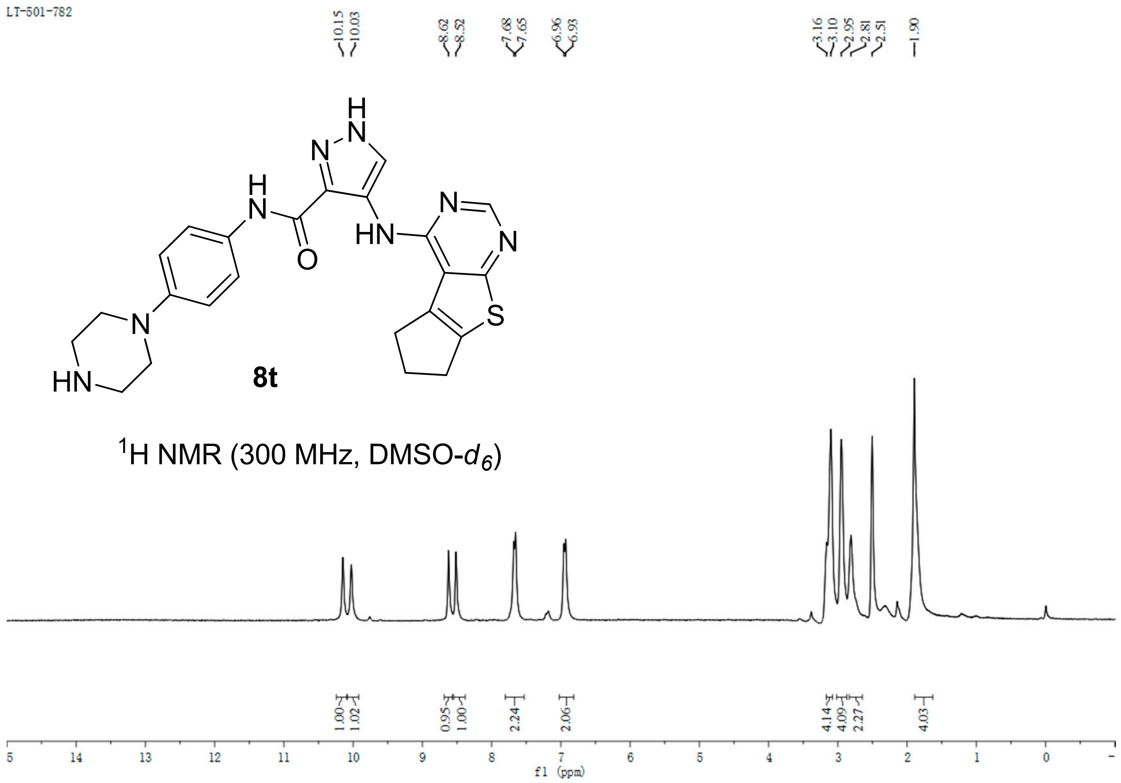
LT-501-782
10.15
10.03
8.62
8.52
7.68
7.65
6.96
6.93
3.16
3.10
2.95
2.81
2.51
1.90
8t
1H NMR (300 MHz, DMSO-d6)
1.00
1.02
0.95
1.00
2.24
2.06
4.14
4.09
2.27
4.03
f1 (ppm)

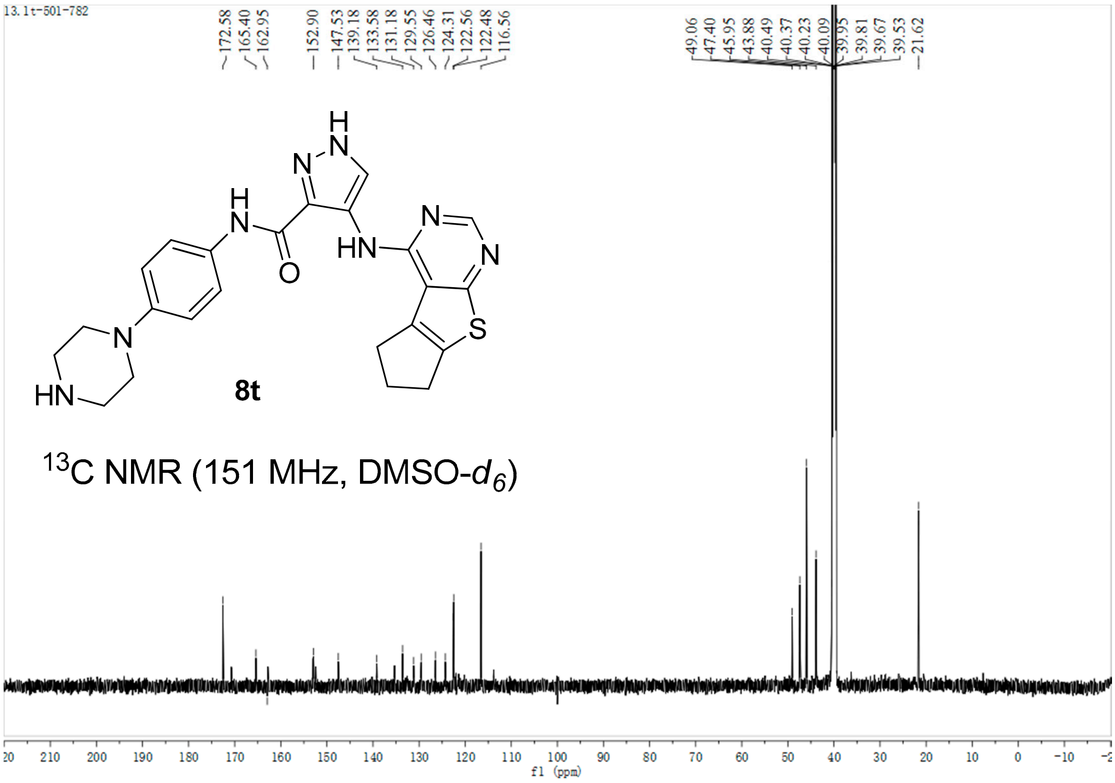
13.1t-501-782
172.58
165.40
162.95
152.90
147.53
139.18
133.58
131.18
129.55
126.46
124.31
122.56
122.48
116.56
49.06
47.40
45.95
43.88
40.49
40.37
40.23
40.09
39.95
39.81
39.67
39.53
21.62
8t
13C NMR (151 MHz, DMSO-d6)
f1 (ppm)
